# Transcriptomics of cumulus cells – a window into oocyte maturation in humans

**DOI:** 10.1186/s13048-020-00696-7

**Published:** 2020-08-12

**Authors:** Brandon A. Wyse, Noga Fuchs Weizman, Seth Kadish, Hanna Balakier, Mugundhine Sangaralingam, Clifford L. Librach

**Affiliations:** 1 CReATe Fertility Centre, 790 Bay St. Suite 420, Toronto, ON M5G 1N8 Canada; 2 Department of Obstetrics and Gynecology; Faculty of Medicine, University of Toronto, Toronto, Canada; 3 Department of Physiology; Faculty of Medicine, University of Toronto, Toronto, Canada; 4 Department of Obstetrics and Gynecology, Women’s College Hospital, Toronto, Canada

**Keywords:** Cumulus cells, Cumulus-oocyte complex, Gene expression, Oocyte maturation, Assisted reproductive technology

## Abstract

**Background:**

Cumulus cells (CC) encapsulate growing oocytes and support their growth and development. Transcriptomic signatures of CC have the potential to serve as valuable non-invasive biomarkers for oocyte competency and potential. The present sibling cumulus-oocyte-complex (COC) cohort study aimed at defining functional variations between oocytes of different maturity exposed to the same stimulation conditions, by assessing the transcriptomic signatures of their corresponding CC. CC were collected from 18 patients with both germinal vesicle and metaphase II oocytes from the same cycle to keep the biological variability between samples to a minimum. RNA sequencing, differential expression, pathway analysis, and leading-edge were performed to highlight functional differences between CC encapsulating oocytes of different maturity.

**Results:**

Transcriptomic signatures representing CC encapsulating oocytes of different maturity clustered separately on principal component analysis with 1818 genes differentially expressed. CCs encapsulating mature oocytes were more transcriptionally synchronized when compared with CCs encapsulating immature oocytes. Moreover, the transcriptional activity was lower, albeit not absent, in CC encapsulating mature oocytes, with 2407 fewer transcripts detected than in CC encapsulating immature (germinal vesicle - GV) oocytes. Hallmark pathways and ovarian processes that were affected by oocyte maturity included cell cycle regulation, steroid metabolism, apoptosis, extracellular matrix remodeling, and inflammation.

**Conclusions:**

Herein we review our findings and discuss how they align with previous literature addressing transcriptomic signatures of oocyte maturation. Our findings support the available literature and enhance it with several genes and pathways, which have not been previously implicated in promoting human oocyte maturation. This study lays the ground for future functional studies that can enhance our understanding of human oocyte maturation.

## Background

Cumulus cells (CC) provide somatic support to the maturing oocyte, and together they comprise the functional unit known as Cumulus-Oocyte-Complex (COC) [1–4]. Understanding oocyte maturation and associated pathologies can help improve fertility treatments, as well as culture media conditions in the lab. Cumulus cells can be collected without compromising the oocyte, and their transcriptomic signatures are valuable non-invasive biomarkers for processes within the oocyte [5, 6].

Oocyte maturation is contingent on rapid transcription and translation, governed by paracrine and autocrine signaling prior to ovulation [7–9]. Once matured, the MII oocyte is less transcriptionally active than its precursors, relying on stored mRNA transcripts that were acquired throughout its maturation, to undergo successful fertilization and support early embryo development until embryonic genome activation [10–12]. Moreover, in addition to the stored transcripts, there is active transportation of transcripts from cumulus cells to the growing oocyte through trans-zonal projections [13, 14]. These projections are critical for both oocyte and cumulus cell differentiation [15].

Currently, oocyte assessment relies mainly on morphological criteria that provide little insight on oocyte quality and competence [16]. Furthermore, available techniques for maturing oocytes in-vitro are inefficient and do not provide good alternatives in cases were pathologies of oocyte maturation lead to retrieval of multiple immature eggs despite adequate controlled ovarian stimulation. This is why molecular investigation of processes in COCs responsible for nuclear and cytoplasmic maturation, as well as pathologies that could arise, are key in improving patient treatment and outcomes. The objectives of this study were: 1) to profile the transcriptome of CC from mature MII and immature GV oocytes, from the same treatment cycle, 2) to use the above profile to validate existing transcriptomic literature exploring human oocyte maturation, and 3) to provide a comprehensive list of genes impacted by in vitro maturation which can be used for future studies exploring human oocyte maturation.

## Results

### Collected samples and patient demographics

A total of 22 cumulus-oocyte complexes (COCs) were collected from 11 patients, for RNA sequencing, with a mean age of 33.9 ± 1.6 years old, mean BMI of 25.4 ± 2.3 kg/m^2^, mean Anti-Mullerian Hormone (AMH) levels of 29.8 ± 12.5 pmol/L, mean Day 3 FSH levels of 7.9 ± 1.4 mIU/mL, and the mean number of collected oocytes was 10.6 ± 1.9 (Table 1). All samples had sufficient number of sequencing reads, high average quality scores, and high sequence alignment rates sufficient for differential gene expression analysis, as per guidelines previously published for quality control of RNAseq experiments [17] (Supplemental Table S2).

**Table 1:** Patient/treatment characteristics and IVF lab outcomes

	Mean ± SEM
Age (years)	33.9 ± 1.6
BMI (kg/m^2^)	25.4 ± 2.3
FSH on Day 2/3 (mIU/ml)	7.9 ± 1.5
AFC	13.0 ± 2.2
Days of Stimulation	10.7 ± 0.7
E2 on Trigger (pmol/l)	7395.2 ± 1094.1
LH on Trigger (IU/ml)	3.9 ± 0.1
# Oocytes Retrieved	10.6 ± 1.9
Maturation Rate (%)	67.5 ± 4.6
Fertilization Rate (%)	79.7 ± 4.8
Cleavage Rate (%)	91.7 ± 3.3
Blastulation Rate (%)	47.2 ± 8.2

*AFC* antral follicle count, *AMH* anti-Mullerian hormone, *BMI* body mass index, *E2* estradiol, *FSH* follicle stimulation hormone, *LH* luteinizing hormone

### Samples clustered according to the degree of maturity of the encapsulated oocyte

**Fig. 1:**
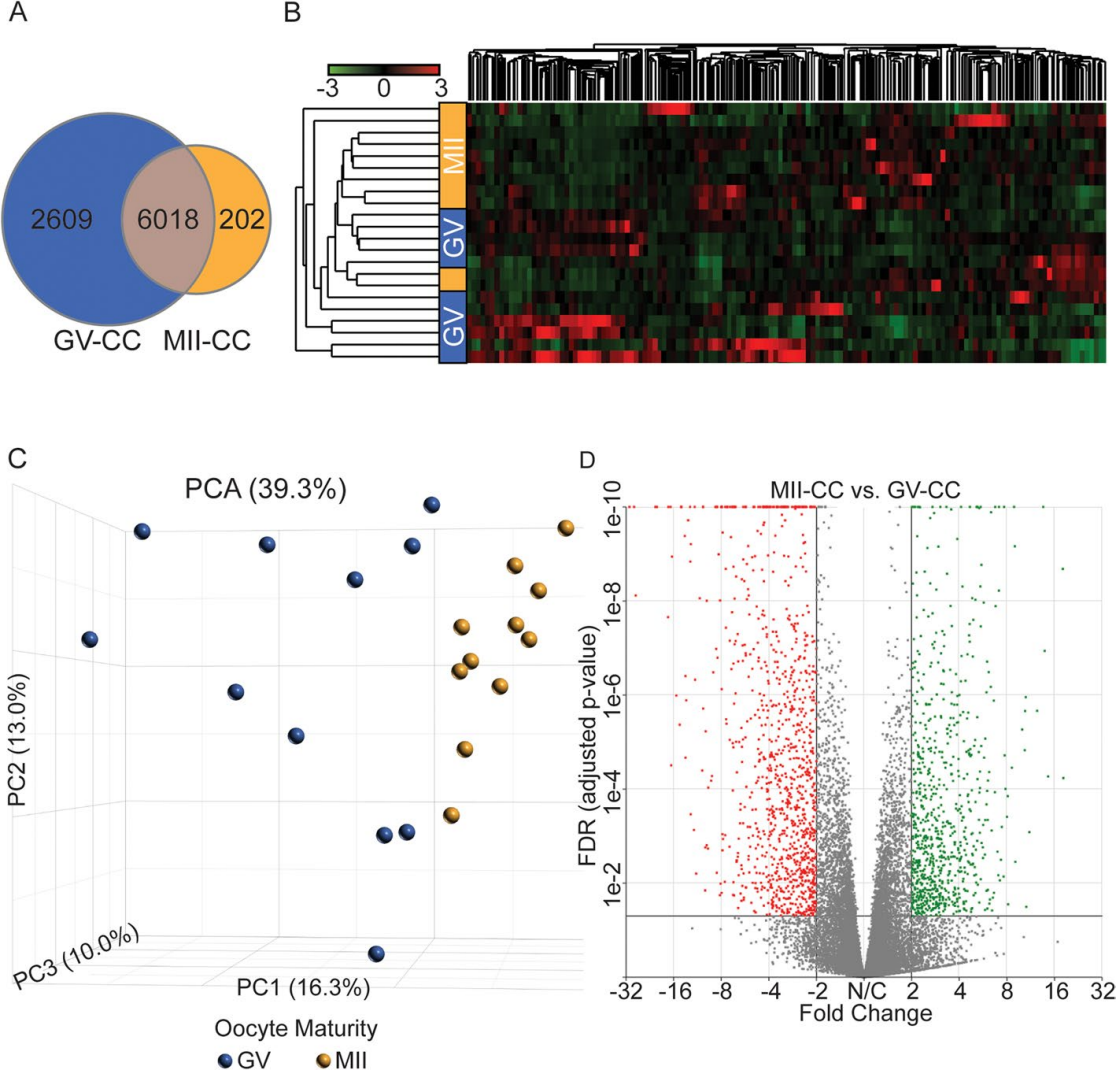
Hierarchical Clustering (HC), Principal Component Analysis (PCA), and Differential Expression (DE) analysis of cumulus cells surrounding mature eggs (MII-CC) compared with cumulus surrounding immature eggs (GV-CC). **a** A Venn diagram depicting the allocation of total number of genes that were expressed in our study. The overlap represents genes commonly expressed in MII-CC (orange) and GV-CC (blue), and genes unique to one of the cohorts in the periphery. **b** Samples cluster by corresponding egg maturity under unsupervised HC. The samples are on rows, and the transcripts are on columns, red indicating upregulated expression and green indicating downregulated expression. **c** PCA of all CC samples shows significant separation along PC1 by oocyte maturity and no apparent effect of patient age, depicted by the size of the sphere. **d** DE analysis between MII-CC and GV-CC using DESeq2; 1818 genes were differentially expressed (1031 downregulated, in red (FC < -2 and FDR < 0.05), and 787 upregulated, in green (FC > 2 and FDR < 0.05))

A total of 6220 genes were detected in the MII-CC cohort, 202 of which were unique, and 8627 genes were detected in the GV-CC cohort, 2609 of which were unique (Fig. 1a). Unsupervised hierarchical clustering demonstrated a separation of the GV-CC cohort from the MII-CC cohort. Notably, the MII-CC cohort clustered more tightly than the GV-CC cohort, indicating that with maturation there is decreased inter-sample variability (Fig. 1b). This was further demonstrated by principal component analysis (PCA) (Fig. 1c) with 16.3% of variability in the dataset corresponding to oocyte maturity (PC1).

### Differential expression reveals marked differences in gene expression according to degree of maturity of the encapsulated oocyte

In order to ensure the sample size and sequencing depth were sufficient to capture biologically significant differences between the MII and GV CCs, a post-hoc power analysis was conducted showing that the minimum required biological replicates is 6 and the minimum sequencing depth is 10 million reads per replicate, both of which were exceeded in this study [18]. When comparing MII-CC with GV-CC cohorts, 1818 genes were differentially expressed (Supplemental Table S3), which comprise 10.3% of annotated RefSeq genes (2 < FC < − 2 and FDR < 0.05). Of these, 40 genes changed by 10-fold or more, 207 genes changed by 5 to 10-fold, and the rest (1571 genes) changed by 2 to 5-fold. When accounting for the direction of differential expression, 1031 genes were significantly downregulated (5.9% of annotated genes) and 787 genes were significantly upregulated (4.5% of annotated genes) in the MII-CC cohort compared with GV-CC (Fig. 1d). The top 20 genes enriched in the different maturation stages are reported in Table 2, all differentially expressed genes are reported in Supplemental Table S3.

**Table 2:** The most abundant genes in the MII-CC and GV- CC cohorts

Enriched in MII-CC Cohort			Enriched in GV-CC Cohort		
Gene Symbol	FDR	FC	Gene Symbol	FDR	FC
*ENPP3*	1.15E-07	13.87	*VIT*	1.48E-11	−30.42
*ACVRL1*	2.50E-16	13.59	*GLRA2*	3.36E-11	−28.47
*ALAS2*	8.23E-04	11.09	*SPRR2E*	2.40E-17	−20.88
*SERF2-C15ORF63*	3.20E-02	10.61	*SFRP4*	1.60E-26	−20.39
*ALDH1A3*	1.11E-06	10.54	*ASB9*	2.20E-08	−17.35
*SLCO4C1*	2.18E-06	10.44	*SPRR2F*	7.50E-30	−17.3
*MMP28*	3.53E-03	9.05	*COL9A1*	1.47E-22	−16.71
*HDC*	6.85E-10	8.97	*GABRA3*	3.12E-05	−16.57
*LPAR3*	8.89E-16	8.88	*NOX4*	1.14E-09	−16.38
*LOC101926963*	9.90E-05	7.84	*LOC105372441*	1.03E-06	−15.37
*SIGLEC1*	1.92E-05	7.74	*MMP20*	4.28E-06	−14.65
*LRRN3*	1.59E-15	7.68	*KLK3*	3.63E-11	− 14.37
*SLC38A8*	7.26E-03	7.63	*CDH3*	2.24E-17	−14.26
*LGALS12*	6.37E-04	7.43	*LRRC2*	4.15E-10	−13.61
*CELA2B*	3.18E-03	7.34	*THEM5*	3.95E-07	−13.47
*CD200R1L*	8.72E-03	7.22	*RHOV*	2.15E-05	−13.46
*CA12*	5.73E-11	7.14	*LEFTY1*	1.29E-06	−13.31
*FHDC1*	6.02E-09	7.12	*DRP2*	1.93E-10	−12.95
*S1PR4*	6.78E-03	7.06	*TDGF1*	5.54E-11	−12.59
*LOC729870*	2.14E-03	6.97	*CLEC18A*	1.46E-09	−12.53

The fold change (FC) is the difference in expression between MII-CC and GV-CC cohorts. The false discovery rate (FDR) represents the statistical strength of each difference

### Novel findings from this study enhance available literature exploring processes that lead to synchronized oocyte maturity

When comparing our differentially expressed genes to those previously reported in the literature, 42 genes were associated with oocyte maturation in both this study and in previous literature (Table 3). Forty-five genes previously correlated with oocyte maturation were not differentially expressed in the current study (Supplemental Table S4) [19]. Three thousand five hundred and fifty-four genes were differentially expressed in our study and have not been annotated previously in studies exploring oocyte maturation. Of these, 129 are known to be regulated by at least one gonadotropin, 82 have been previously reported to be regulated by LH alone, 16 by FSH alone and 31 by both LH and FSH (Supplemental Table S5).

**Table 3:** Potential oocyte maturation biomarkers

Gene ID	Description	Previous Study	Method of Detection	Fold Change in this study
*ADAMTS1*	ADAM Metallopeptidase with Thrombospondin Type 1 Motif 1	Devjak et al. 2012 [20]	RNAseq	2.27
		Yerushalmi et al. 2014 [21]	RNAseq	
*ANK2*	Ankyrin 2	Devjak et al. 2012 [20]	RNAseq	− 3.13
*ANKRD57*	aka. *SOWAHC*, Sosondowah Ankyrin Repeat Domain Family Member C	Ouandaogo et al. 2011 [22]	Microarray	−2.21
*AOC2*	Amine Oxidase, Copper Containing 2	Ouandaogo et al. 2011 [22]	Microarray	3.72
*AREG*	Amphiregulin	Feuerstein et al. 2007 [23]	RT-qPCR	5.4
*BDNF*	Brain Derived Neurotrophic Factor	Anderson et al. 2009 [24]	RT-qPCR	2.68
*BMP2*	Bone Morphogenetic Protein 2	Devjak et al. 2012 [20]	RNAseq	2.46
*BUB1*	BUB1 Mitotic Checkpoint Serine/Threonine Kinase	Devjak et al. 2012 [20]	RNAseq	−4.28
		Feuerstein et al. 2012 [25]	Microarray	
*C10orf10*	aka. *DEPP1*, Autophagy Regulator	Devjak et al. 2012 [20]	RNAseq	2.99
*CCDC99*	aka. *SPDL1*, Spindle Apparatus Coiled-Coil Protein 1	Devjak et al. 2012 [20]	RNAseq	− 3.46
*CDH3*	Cadherin 3	Devjak et al. 2012 [20]	RNAseq	−14.26
*COX2*	aka. *PTGS2*, Prostaglandin-Endoperoxide Synthase 2	Feuerstein et al. 2007 [23]	RT-qPCR	4.00
		Anderson et al. 2009 [24]	RT-qPCR	
		Wathlet et al. 2011 [26]	RT-qPCR	
		Yerushalmi et al. 2014 [21]	RNAseq	
*CRHBP*	Corticotropin Releasing Hormone Binding Protein	Devjak et al. 2012 [20]	RNAseq	−5.41
*DHCR24*	24-Dehydrocholesterol Reductase	Yerushalmi et al. 2014 [21]	RNAseq	2.29
*DSE*	Dermatan Sulfate Epimerase	Devjak et al. 2012 [20]	RNAseq	−2.36
*F2RL1*	F2R Like Trypsin Receptor 1	Ouandaogo et al. 2011 [22]	Microarray	−3.06
*FSHR*	Follicle Stimulating Hormone Receptor	Yerushalmi et al. 2014 [21]	RNAseq	−8.13
*GABRA5*	Gamma-Aminobutyric Acid Type A Receptor Alpha5 Subunit	Devjak et al. 2012 [20]	RNAseq	− 3.93
*GLRA2*	Glycine Receptor Alpha 2	Devjak et al. 2012 [20]	RNAseq	−28.47
*GPX*	Glutathione Peroxidase 3	Yerushalmi et al. 2014 [21]	RNAseq	−3.56
*GREM1*	Gremlin 1, DAN Family BMP Antagonist	Anderson et al. 2009 [24]	RT-qPCR	−2.03
		Yerushalmi et al. 2014 [21]	RNAseq	
*HSD11B1*	Hydroxysteroid 11-Beta Dehydrogenase 1	Devjak et al. 2012 [20]	RNAseq	2.95
*ID2*	Inhibitor of DNA Binding 2	Ouandaogo et al. 2011 [22]	Microarray	3.53
*ID3*	Inhibitor of DNA Binding 3	Devjak et al. 2012 [20]	RNAseq	− 4.9
*ITGB3*	Integrin Subunit Beta 3	Devjak et al. 2012 [20]	RNAseq	− 4.05
*ITPKA*	Inositol-Trisphosphate 3-Kinase A	Wathlet et al. 2011 [26]	RT-qPCR	2.49
*LHCGR*	Luteinizing Hormone/Choriogonadotropin Receptor	Yerushalmi et al. 2014 [21]	RNAseq	3.72
*MAOB*	Monoamine Oxidase B	Devjak et al. 2012 [20]	RNAseq	− 2.38
*MGP*	Matrix Gla Protein	Devjak et al. 2012 [20]	RNAseq	− 8.01
*NDP*	Norrin Cystine Knot Growth Factor	Devjak et al. 2012 [20]	RNAseq	− 2.4
*NID2*	Nidogen 2	Devjak et al. 2012 [20]	RNAseq	5.46
*NKAIN1*	Sodium/Potassium Transporting ATPase Interacting 1	Devjak et al. 2012 [20]	RNAseq	4.38
*NOS2*	Nitric Oxide Synthase 2	Yerushalmi et al. 2014 [21]	RNAseq	−2.48
*PALLD*	Palladin, Cytoskeletal Associated Protein	Devjak et al. 2012 [20]	RNAseq	−4.13
*PTX3*	Pentraxin 3	Zhang et al. 2005 [27]	Microarray	3.08
		Anderson et al. 2009 [24]	RT-qPCR	
*SERPINE2*	Serpin Family E Member 2	Feuerstein et al. 2012 [25]	Microarray	− 4.31
		Yerushalmi et al. 2014 [21]	RNAseq	
*SFRP4*	Secreted Frizzled Related Protein 4	Devjak et al. 2012 [20]	RNAseq	− 20.39
		Feuerstein et al. 2012 [25]	Microarray	
		Yerushalmi et al. 2014 [21]	RNAseq	
*SPOCK2*	SPARC (Osteonectin), Cwcv And Kazal Like Domains Proteoglycan 2	Devjak et al. 2012 [20]	RNAseq	2.88
		Feuerstein et al. 2012 [25]	Microarray	
*STAR*	Steroidogenic Acute Regulatory Protein	Feuerstein et al. 2007 [23]	RT-qPCR	2.67
		Yerushalmi et al. 2014 [21]	RNAseq	
*TLL2*	Tolloid Like 2	Yerushalmi et al. 2014 [21]	RNAseq	−3.17
*TNFSF4*	TNF Superfamily Member 4	Devjak et al. 2012 [20]	RNAseq	−4.01
*TSPAN7*	Tetraspanin 7	Devjak et al. 2012 [20]	RNAseq	−3.3

### Pathway analysis and leading-edge analysis revealed significant differences between maturational stages

**Fig. 2:**
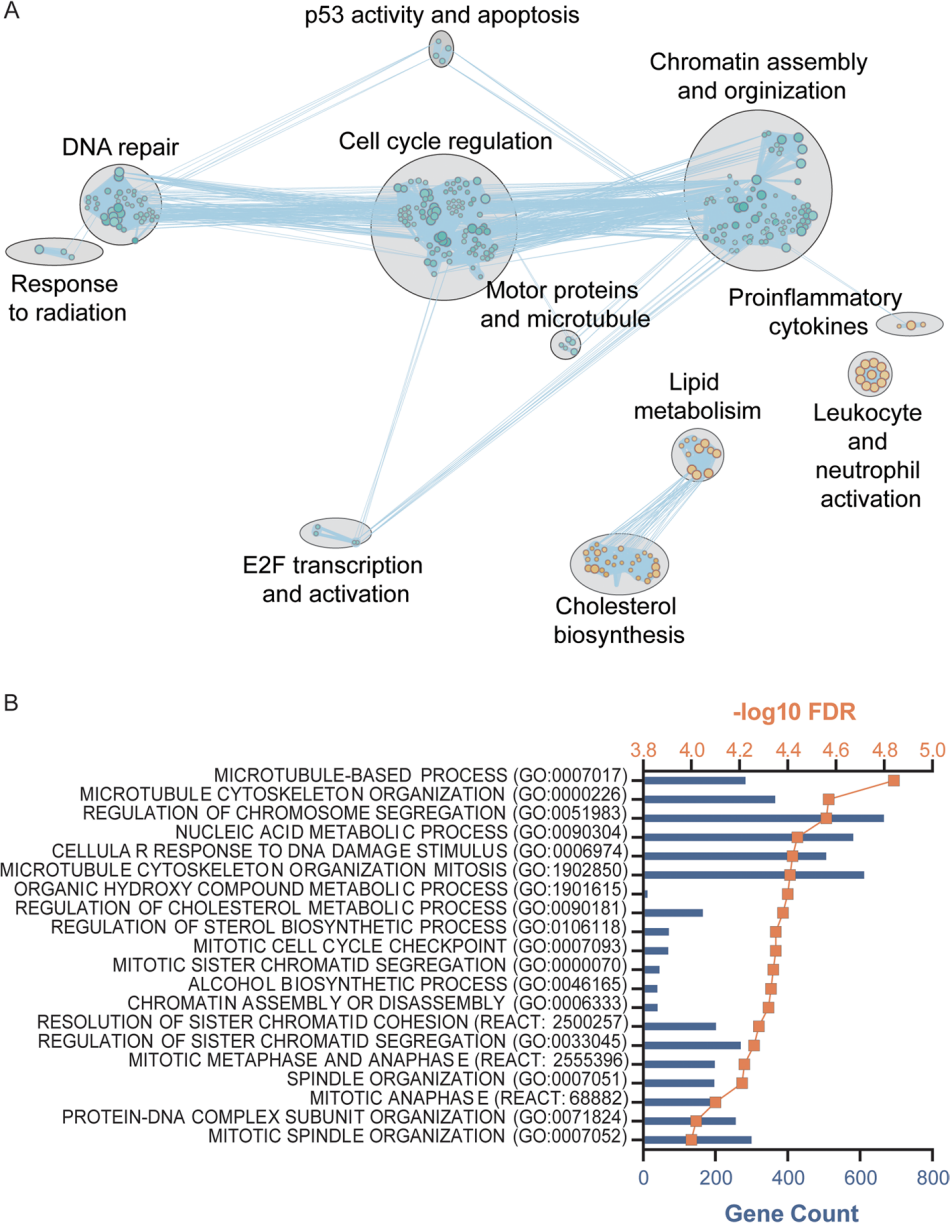
Pathway analysis of significantly differentially expressed genes. GSEA (Gene Set Enrichment Analysis) reveals that the MII-CC cohort significantly downregulate pathways involved in chromatin assembly, apoptosis, mitotic cell cycle control, and DNA repair processing (in blue) and significantly upregulate pathways involved in lipid biosynthesis, steroid metabolism, inflammation, and leukocyte activation (in orange). A total of 60 gene sets were enriched in upregulated genes and 223 gene sets were enriched in downregulated genes at FDR q-value < 0.05. The size of the node corresponds to the number of genes in each gene set

Gene set enrichment analysis (GSEA) was conducted to determine the pathways and cellular processes that are altered throughout oocyte maturity, thus allowing for the interpretation of the complex interactions between differentially expressed genes (Fig. 2) [28]. Furthermore, leading edge analysis (LEA) helped determine which genes were driving pathway enrichment scores, and to better understand the major biological differences between the two cohorts.

#### Pathways and processes that were primarily enriched in downregulated genes in the MII-CC cohort included

**Fig. 3:**
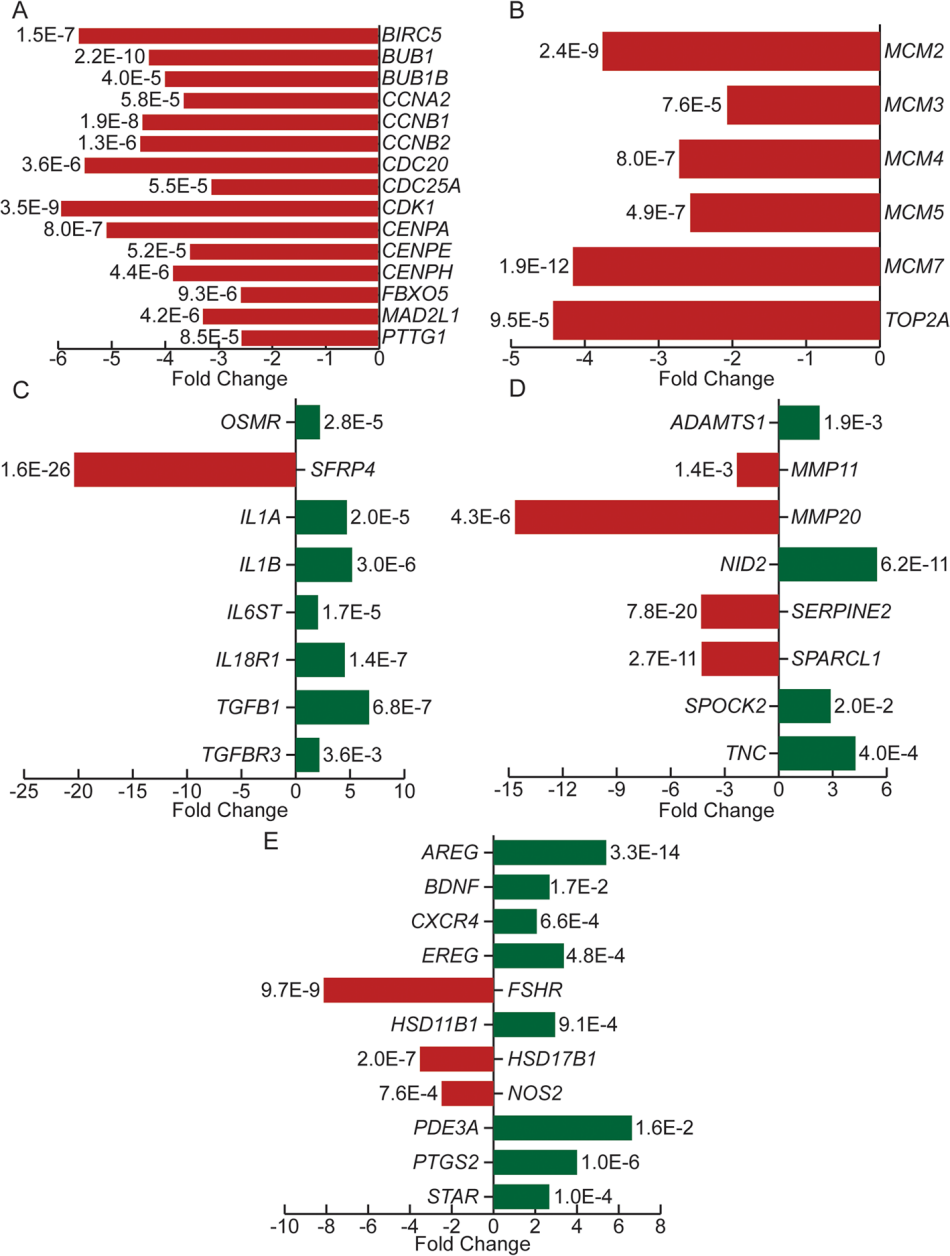
Differential expression of hallmark genes involved in the major pathways and processes identified by GSEA, leading edge analysis (LEA), and/or have been previously implicated as important for oocyte maturation. **a** Nuclear maturation; **b** Chromatin remodeling and DNA replication initiation; **c** Apoptosis and inflammation; **d** Extracellular matrix components and remodeling; **e** Steroid metabolism and processing. Red indicates significantly downregulated genes and green indicates significantly upregulated genes in MII-CC compared with GV-CC. FDR is reported beside each bar

Nuclear maturation, chromatin remodeling and replication initiation, faithful chromosome segregation, apoptosis and inflammation (Fig. 2). Furthermore, specific genes identified as biologically significant genes for oocyte maturation including nuclear maturation (Fig. 3a), chromatin remodeling and DNA replication initiation (Fig. 3b), and apoptosis and inflammation (Fig. 3c) are further highlighted in Fig. 3.

#### Pathways and processes that were primarily enriched in upregulated genes in the MII-CC cohort included

Extracellular matrix (ECM) components and its remodeling enzymes, and steroid metabolism and processing (Fig. 2). Genes identified as biologically significant genes for oocyte maturation including ECM remodeling (Fig. 3d), and steroid metabolism (Fig. 3e) are further highlighted in Fig. 3.

Leading edge analysis identified several genes involved in cell cycle control (*CDK1, CCNB1, CCNB2, CCNA2, BUB1,* and *CDC20*), DNA replication initiation (*MCM2–7*), and centromere assembly and organization (*CENPF*), among others, as the genes that were most significantly driving the gene set enrichment analysis (Supplemental Table S6). GSEA on DE genes known to be regulated by at least one gonadotropin revealed two major pathways; transcriptional regulation of tp53 (apoptosis), overall enriched in downregulated genes, and metabolic biosynthesis, overall enriched in upregulated genes. LEA identified several genes involved in cell cycle control and cell death indicating that the MII-CC cohort, in response to LH and FSH decreases cell death signaling and increases biosynthesis.

### Validation of NGS results by qPCR

**Fig. 4:**
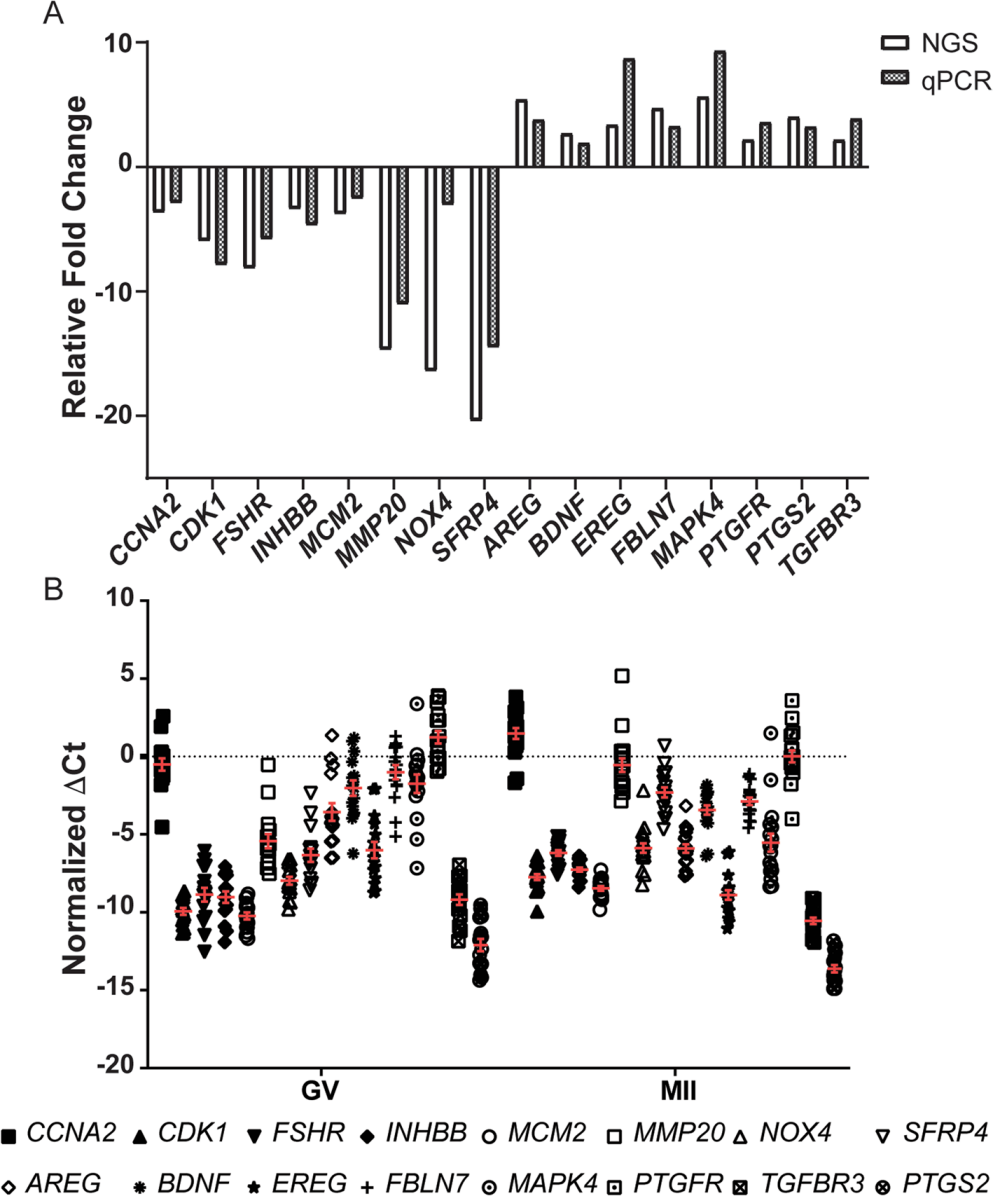
**a** Validation of RNAseq results by qPCR of 16 targets and normalized to *RPLP0* in duplicate. Fold change was calculated using the ∆∆Ct method between the MII-CC and GV-CC cohorts. Results of RNAseq (open bars) and qPCR (filled bars) are presented as fold change between MII-CC and GV-CC samples. **b** Additional 34 CC from 7 patients (18 MII-CC and 16 GV-CC) analyzed using qPCR for 17 targets (16 genes and one reference gene *RPLP0*), in duplicate. Normalized ∆Ct values are plotted for each sample, horizontal line represents mean ∆Ct, and error bars represent SEM

We selected 16 differentially expressed genes as determined by RNAseq and are known to be involved in various pathways of CC expansion and oocyte maturation. For all selected genes, similar fold changes were observed using qPCR as were observed using NGS (Fig. 4a). In addition, when assessing multiple CCs from 7 additional patients at both the MII and GV stage (from 2/3 GV-CC and 2/3 MII-CC per patient) (a total of 34 COCs), the expression of all tested genes was consistent within each patient. Interestingly, the expression of all genes used for validation across all MII-CC were consistent both within and between patients (∆Ct SEM (range) = 0.26(0.12–0.56)). GV-CC were also consistent within and between patients, however to a lesser extent (∆Ct SEM (range) = 0.40(0.21–0.57)) (Fig. 4b).

This was further validated using Pearson’s R correlation which also demonstrated that the cohort with the highest similarity was MII-CC within the same patient, with a R^2^ of 0.954 ± 0.023 followed by GV-CC within the same patient, with a R^2^ of 0.906 ± 0.036. The correlation coefficients and significance are outlined in Table 4. Taken together, this indicates that the heterogeneity of the population of CCs within the ovary is low, and sampling one mature MII-CC and one immature GV-CC for NGS is representative of the cohort of CCs.

**Table 4:** Similarities between points was measured by creating an overall correlation between the dCt expression of all 16 target genes using SPSS Proximities

	Correlation (R^**2**^) and SD	Significance
Overall	.821 (.141)	–
Overall GV-CC	.883 (.083)	t = 6.94 *p* = 0.00004^*‡^
Overall MII-CC	.936 (.037)	
Within GV-CC individual	.906 (.039)	t = 2.35 *p* = 0.0567^#^
Within MII-CC individual	.954 (.023)	

## Discussion

This study is novel in the choice of cohorts for comparison. We included CC encapsulating oocytes arrested at the GV stage, despite being exposed to adequate stimulation, and compared them with CC encapsulating MII oocytes that matured in vivo, from the same patients during an IVF cycle. This comparison allowed us to fine-tune our understanding of oocyte maturation in-vivo. Previous human oocyte maturation studies analyzed COCs from in-vitro maturation cycles [16, 21, 29–32]. This is why their findings are more relevant for processes in in-vitro maturation per se, but they do not tease out factors specifically associated with failed maturation despite adequate in-vivo exposure to controlled ovarian hyper stimulation (COH).

Forty-two differentially expressed genes in our study, have been previously associated with oocyte maturation and cumulus cell expansion in IVF treatments (Table 3). Following our extensive literature search, we identified 45 genes, which were previously correlated with human oocyte maturation, but not captured by our study design, possibly because they may impact IVM alone and may not reflect in vivo maturation (Supplemental Table S4). A third group of 3554 genes captured in the current study but not in previous studies which represents a novel group of genes that should be further explored as they have not been previously implicated in human oocyte maturation (Supplemental Table S3). Of these, 129 genes have been previously shown to be regulated by either LH, FSH, or both (Supplemental Table S5).

In this study, several factors and their regulators involved in nuclear maturation and cell cycle control were differentially expressed between cumulus cells encapsulating oocytes of different maturity, reiterating findings from previous studies [21, 22, 33, 34]. These include cell cycle regulators (*BIRC5, BUB1, BUB1B, CCNA2, CCNB, CDK1, FBXO5 MAD2L1,* and *PTTG1*) and components of the centromere (*CENPA, CENPE,* and *CENPH*) [21]. In our MII-CC cohort we observed downregulation of *MCM2–7*, which form the hexameric pre-replication protein complex. This complex is involved in initiating replication forks and recruiting other DNA replication related proteins. We also observed downregulation of *TOP2A*, which relaxes supercoiled and circular DNA molecules. Reinforcing available literature that states that while crucial at the MI stage for chromatin remodeling [35, 36], its activity decreases in mature oocytes [37].

Apoptosis was also attenuated in the MII-CC cohort, further supporting decreased cell turnover with advanced maturity. Related pathways including Wnt pathway and Akt-pathway were affected, as demonstrated by downregulation of *SFRP4*, a potent inhibitor of Wnt signaling [38], and upregulation of *OSMR*, an activator of Akt-mediated proliferation [39]. These findings corroborate previous literature reporting downregulation of *SFRP4* during oocyte maturation [20, 40, 41], and upregulation of *OSMR* in bovine preovulatory follicles post-triggering by gonadotropins [42].

Extracellular matrix remodeling was also altered between the two maturity cohorts, as evident by members of the matrix metalloproteinases (MMP) family and their inducers (*MMP11* and *SPARC1L*). Again, this supports previous literature showing significant decrease of *MMP11* in granulosa cells following hCG administration [43]. This effect is further demonstrated by increased expression of *TNC*, *NID2,* and *SPOCK2* - all ECM proteins and MMP substrates [20, 44–46]. Notably, well characterized ECM remodeling enzymes, *ADAMTS1* and *SERPINE2*, were also differentially expressed, aligning with previous studies [47, 48]. Both play critical roles in follicular remodeling during follicular growth and rupture [49], by metabolizing Versican and Hyaluronan which lead to cumulus cell matrix expansion and attenuation [50].

Another key process enhanced in follicular niche maturation is inflammation, which is crucial for ovulation. Upon gonadotropin stimulation, the follicle wall is weakened, thereby facilitating its eventual rupture [51]. In our MII-CC cohort, we observed marked upregulation of genes associated with inflammation, including members of the Interleukin and TGF-beta families. Among the genes upregulated in our MII-CC cohort were *IL18R1* which promotes cumulus cell expansion [52], and *TGFBR3* which promotes cellular differentiation, migration, adhesion and extracellular matrix production [53, 54]. *IL6ST* which is part of the cytokine receptor complex (gp130) was also upregulated in the MII-CC cohort, consistent with previous studies in non-human primates and equine models [55, 56].

Key players that emerged in our cohort as being significant for cumulus cells to facilitate oocyte maturation are *AREG, EREG, PTGS2,* and *STAR*. Two factors at the heart of this complex process are *AREG* and *EREG,* which have been shown to mediate the LH signal driving cumulus expansion and oocyte maturation [31, 33, 57]. They also activate the EGF receptor (EGFR) which in turn releases matrix metalloproteinases (MMPs) and promotes cumulus expansion [57, 58]. Furthermore, in conjunction with progesterone, *AREG* and *EREG* enhance *PTGS2* (also upregulated in our MII-CC cohort) via EGF to increase prostaglandin production and maintenance of chromosomal spindles [32, 59–61]. In addition, *AREG* mediates hCG-induced *STAR* expression (also upregulated in our MII-CC cohort), which plays a key role in steroid and progesterone production in human granulosa cells [62], and is a potential predictive biomarker for nuclear maturation [23] and oocyte quality [32]. It is important to note, that despite being well defined as key in ovarian maturation [31, 57, 63], *EREG* has not been found to be differentially expressed in previous genomic signature studies addressing this question. This further highlights the importance of our study design in better refining the pathophysiology of oocyte maturation.

Other critical factors confirmed by this study are *IL1, FSHR, BDNF, HSD11B1,* and *HSD17B1,* all of which are implicated in the control of steroid synthesis and paracrine response to steroids.

*IL1* (both alpha and beta subunits), which stimulates steroidogenesis, was upregulated in the MII-CC cohort with a concurrent decreased expression of *FSHR* in the same cohort, substantiating what was previously observed in rodents and humans [64, 65]. *BDNF*, which modulates granulosa cell function via FSHR-coupled signaling pathway, to affect aromatase-mediated steroidogenesis, was also downregulated in our MII-CC cohort [66].

*HSD11B1,* the enzyme responsible for cortisone production, an essential substrate for steroid hormone synthesis, was upregulated in our MII-CC cohort. A companion enzyme, *HSD17B1,* catalyzes the last step in estrogen metabolism converting E1 of low estrogenic activity to E2 of high activity using cortisone as a substrate [67]. *HSD17B1* has not been captured in previous human studies, but was downregulated in our MII-CC cohort, consistent with the results seen in a previous bovine study [68], and further highlighting the advantage of our study design.

Both LHCGR and FSHR were differentially expressed in the MII-CC cohort when compared to the GV-CC cohort. To further explore how this may impact the transcriptional profiles, we identified all genes differentially expressed between these two cohorts that are known to be regulated by LH and/or FSH. GSEA based on these 129 genes, identified two major pathways: regulation of apoptosis (via p53) and biosynthesis of various biomolecules.

Overall, apoptosis was enriched in downregulated genes. Interestingly, several major players in the regulation of apoptosis, including *BIRC5*, *TP53, HMGB1*, *HMGB2*, and *SFRP4* are also known to be regulated by LH and/or FSH [38, 40, 69–71]. Taken together, these findings suggest that the inability of the GV-CC cohort to appropriately respond to FSH/LH may, in turn, lead to failure of the CCs to effectively dampen the apoptotic signals, causing the COC to enter a stage of maturation arrest and follicular atresia.

Overall, biosynthesis was enriched in upregulated genes among the MII-CC cohort. Notably, several members of the CYP family, which were upregulated, and are involved in the biosynthesis of estrogen and androgens, are known to be regulated by LH and/or FSH [72–74]. Taken together, these findings suggest that the MII-CCs are responding adequately to gonadotropin administration.

Collectively, it appears that the GV-CC cohort, failed to adequately synthesize estrogen, despite exposure to gonadotropins and, thus, began to upregulate genes involved in apoptosis. This may be due to insufficient LH and/or FSH receptors on these COCs or due to another underlying malady.

Finally, we show that *PDE3A*, known to improve nuclear-cytoplasmic synchrony [75], is significantly upregulated in our MII-CC cohort. While this gene has not been studied in cumulus cells in the context of oocyte maturation in humans, it has been shown that an increase in oocyte PDE3A activity causes delayed spontaneous meiotic maturation, coupled with extended gap junctional communication between the CC and the oocyte. Such a delay has a positive effect on oocyte cytoplasmic maturation, thereby improving oocyte developmental potential [76]. The fact that upregulation of this gene was captured by our study design speaks once again to the strength of our study and to what it adds to current literature.

Methodological strengths of this study include (i) a sibling COC design allowing to minimize the biologic variability between cohorts, (ii) exploring transcriptomic dynamics in cumulus cells, which are considered valuable non-invasive markers for oocyte quality [77–79], and (iii) performing next generation sequencing (NGS), which is the most unbiased approach currently available for exploring transcriptomic signatures. A methodological weakness of this study is our inability to compare our findings with a third cohort of CCs encapsulating naïve GVs from the same stimulation cycle. Furthermore, to keep our sequencing sample size small, only 2 cumulus masses were selected from each patient; 1 to represent the immature GV COCs, and the other to represent the mature MII COCs. To lower the risk for selection bias due to this study design, we chose to perform validation studies that established the tight correlations within each cohort. Lastly, our small sample size and study design did not allow for tracking outcomes on an individual oocyte basis.

## Conclusions

In conclusion, our findings enhance current literature on oocyte maturation by identifying CC genes not previously associated with oocyte maturation that may be involved in this process. Our novel list of genes can serve as a springboard for future studies. Our future studies will focus on determining the functional significance of these findings and on attempting to identify how different treatment options may favor a more synchronized mature/competent state. In addition, to further validate genes that are critical for oocyte maturation and competency, further large-scale studies correlating gene expression with clinical outcomes using a targeted transcriptome panel are needed.

## Materials and methods

### Patient recruitment, data collection and cumulus cell isolation

Cumulus cell samples were collected from eighteen patients undergoing IVF-ICSI cycles at the CReATe Fertility Centre (Toronto, ON, Canada), between August 2016 and June 2017. Exclusion criteria were patients diagnosed with PCOS, as per Rotterdam criteria, as well as patients with endometriosis diagnosed by laparoscopy. Samples from eleven patients were used for RNA-seq (22 COCs, 11 mature (MII), and 11 immature (GV)), and samples from seven additional patients were used for qPCR validation of the findings (a total of 18 mature (MII) and 16 immature (GV) COCs). Patients were treated using a standard antagonist protocol, with initial gonadotropin dosing and subsequent adjustments at the discretion of the treating physician.

Ultrasound guided oocyte retrieval was performed 35-36 h post hCG injection. COCs were identified under a stereomicroscope and only COCs completely and tightly enclosed by compact CCs were used for this study to minimize the potential collection of contaminating granulosa cells. Selected COCs were serially washed three times in Quinn’s Advantage Medium (Sage, USA) to remove cellular contaminants, and to further reduce the possibility of granulosa cell contamination. CCs were then mechanically separated from each oocyte individually in Quinn’s Advantage Medium (Sage, USA), under paraffin oil by one experienced embryologist within 1 h of oocyte retrieval. The oocytes corresponding to individually collected CC were separately exposed to hyaluronidase (80 IU/ml) immediately after mechanical separation of CCs, washed in Quinn’s Advantage Medium (Sage, USA). Maturational stage was assessed through the observation of the nucleus of the oocyte. Oocytes with an extruded polar body were deemed mature (MII), oocytes with an intact germinal vesicle was deemed immature (GV), oocytes without an observable germinal vesicle or an extruded polar body were deemed MI and excluded from further analysis. The CC were collected from single MII (*n* = 29) and GV oocytes (*n* = 27), frozen separately in 300ul of RNA lysis buffer RL (Norgen Biotek, Canada), and stored at − 80 °C until RNA extraction. Clinical data including patient demographics, medical history, and ovarian stimulation related parameters, were collected for all patients enrolled in this study.

### RNA extraction and NGS library preparation

RNA extraction, cDNA conversion and NGS library construction and normalization were conducted as previously described [80]. Briefly, for NGS one MII-CC and one GV-CC were chosen at random from each of the 11 patients for RNA extraction using the Total RNA Purification Kit Micro (Norgen Biotek, Canada). The quantity was assessed using Qubit RNA HS (ThermoFisher, USA) and RNA integrity assessed using 2100 Bioanalyser RNA 6000 Pico Total RNA Kit (Agilent Technologies, Canada). cDNA was synthesized using the SMART-seq v4 Ultra Low Input RNA Kit (Takara Bio Inc., Japan) according to the sample preparation guide and using 14 rounds of amplification. Sequencing libraries were constructed using Nextera XT (Illumina, USA) and 1 ng of amplified cDNA according to the sample preparation guide. Final sequencing libraries were assessed for quantity and quality using the KAPA Library Quantification Kit (KAPA Biosystems, Switzerland) and 2100 Bioanalyser High Sensitivity DNA Kit (Agilent Technologies), respectively. Normalized libraries were pooled, denatured, diluted to 1.4 pmol/l and loaded onto a High Output (300 cycle) flow cell (Illumina, USA) followed by sequencing (2 × 127 bp) on a NextSeq 550 (Illumina, USA).

### Bioinformatics

#### Differential expression

The recommendations outlined by Ching et al. 2014 were followed when selecting the differential expression package, as well as using a paired-sample RNA-Seq design as suggested [17]. FASTQ files were generated using bcl2fastq2 (v2.17) and the read quality was assessed. Sequences were trimmed based on quality (Phred > 28). Raw trimmed reads were aligned to Human Genome Assembly 38 (hg38) using STAR (v2.5.3a) [81] and quantified to RefSeq (Release 84). Low expressed transcripts were excluded (maximum counts < 10) and differential expression (DE) was conducted on the remaining counts using DESeq2 (v3.5) [82]. Principal Component Analysis (PCA) and hierarchical clustering (HC) were conducted to assess the relationship between samples and determine covariates contributing to variation in the dataset. Principal component 1 (PC1) accounts for the largest proportion of the variability observed within the dataset, PC2 accounts for the second largest, and so on. Differentially expressed genes were identified by comparing all mature CC samples (MII-CC) to all immature CC samples (GV-CC), and were deemed to be differentially expressed if the gene had a Fold change (FC) of more than absolute value of 2, and a false discovery rate (FDR) < 0.05. This analysis was conducted in Partek Flow (version 8.0.19.0408).

#### Pathway analysis

Gene Set Enrichment Analysis (GSEA) was performed to determine the effect all differentially expressed genes have on cellular processes and functions [28]. The resulting pathway list was cross referenced with a custom gene set created and supported by the Bader Lab (University of Toronto) which is comprised of all GO database, KEGG, and Reactome gene sets (v2018-12-01) (http://download.baderlab.org/EM_Genesets/) [83]. Genes that could not be mapped to any gene-set term were excluded from the comparison. Gene sets with 10 or fewer genes and/or a q-value > 0.05 were excluded from further analysis. Following GSEA, leading edge analysis (LEA) was conducted to determine what genes were driving the gene set enrichment score, as well as to highlight genes that were shared between gene sets.

To further explore the impact FSH and/or LH may have on the transcriptome, we identified all differentially expressed genes that are known to be regulated by LH, FSH or both [84] and performed GSEA and LEA as described previously.

### NGS validation by qPCR

Sixteen genes (and one reference gene) were chosen for validation from the list of differentially expressed genes. The choice of genes was based on previous annotations deeming these genes as biologically significant, identification by leading edge analysis, and/or participation in key ovarian gene pathways. Pre-designed and validated PrimeTime™ qPCR assays (IDT, USA) were used for validation of NGS results with *RPLP0* as the reference gene. All targets were assayed in duplicate using PrimeTime™ Gene Expression MasterMix (IDT, USA) (polymerase activation at 95 °C for 3 min; 45 cycles of 15 s denaturation at 95 °C and 1 min annealing/extension at 60 °C). Relative fold change (ΔΔCt) was employed to quantify gene expression [85]. Data analysis was performed using GraphPad Prism (version 5.02). The list of primers and probes used for validation are given in Supplemental Table S1. qPCR for the above validated genes was also performed on multiple COCs of the same maturational stages on samples from 7 additional patients. This was carried out to ensure the validity of a random choice of a single COC as a representative of all COCs at the same maturational stage from the same patient. Similarities between individual CCs was measured by creating an overall Pearson correlation between the vectors of variables (16 targets genes) using SPSS Proximities. This created an overall Pearson R^2^. Paired t-test was used to compare correlations within GV-CC and within MII-CC groups, separately. A non-paired test was used to compare overall GV-CC and overall MII-CC correlations due to an unequal number of observations for some individuals.

### Gene annotation and literature search

To determine the clinical significance of our bioinformatic findings, differentially expressed genes were cross referenced with available datasets in the literature by searching the PubMed database for previous studies assessing the transcriptome of human CC using NGS, Microarray, or qPCR. Differentially expressed genes were further reviewed in depth using the Ovarian Kaleidoscope Database [84] and GeneCards Human Gene databases (http://www.genecards.org/), to correlate our bioinformatic findings with hallmark physiological and pathological processes in the ovary.

## Supplementary information

**Additional file 1 : Supplemental Table S1:** List of primers and probes used for qPCR validation of RNAseq results. **Supplemental Table S2:** RNAseq quality control metrics for each cohort presented as mean ± SEM (Range). **Supplemental Table S3:** All differentially expressed genes between MII-CC and GV-CC cohorts. **Supplemental Table S4:** Differentially expressed genes known to be regulated by LH, FSH, or both with their associated fold change, and number of gene sets that are found in following leading edge analysis. **Supplemental Table S5:** Previously identified genes implicated in oocyte maturation in vitro that were not differentially expressed in this study. **Supplemental Table S6:** Top 50 genes identified by leading edge analysis ranked by the number of unique gene-sets the gene was identified in. **Supplemental Table S7:** Normalized read counts of all samples.

## Data Availability

The dataset supporting the conclusions of this article is included within the article (Supplementary Table S7).
