# Correction: Transcriptomics of cumulus cells – a window into oocyte maturation in humans

**DOI:** 10.1186/s13048-024-01529-7

**Published:** 2024-11-15

**Authors:** Brandon A. Wyse, Noga Fuchs Weizman, Seth Kadish, Hanna Balakier, Mugundhine Sangaralingam, Clifford L. Librach

**Affiliations:** 1 CReATe Fertility Centre, 790 Bay St. Suite 420, Toronto, ON M5G 1N8 Canada; 2 Department of Obstetrics and Gynecology, Faculty of Medicine, University of Toronto, Toronto, Canada; 3 Department of Physiology, Faculty of Medicine, University of Toronto, Toronto, Canada; 4 Department of Obstetrics and Gynecology, Women’s College Hospital, Toronto, Canada

**Correction** **:** **J Ovarian Res 13, 93 (2020)**

**https://doi.org/10.1186/s13048-020-00696-7**

Following publication of the original article [1], the authors reported the below errors:

1. Reference citations in table 3 were incorrect. Correct table 3 is shown below.

Incorrect Table 3:

**Table 3:** Potential oocyte maturation biomarkers

**Gene ID**	**Description**	**Previous Study**	**Method of Detection**	**Fold Change in this study**
*ADAMTS1*	ADAM Metallopeptidase with Thrombospondin Type 1 Motif 1	Devjak et al. 2012 [19]	RNAseq	2.27
		Yerushalmi et al. 2014 [20]	RNAseq	
*ANK2*	Ankyrin 2	Devjak et al. 2012 [19]	RNAseq	− 3.13
*ANKRD57*	aka. *SOWAHC*, Sosondowah Ankyrin Repeat Domain Family Member C	Ouandaogo et al. 2011 [21]	Microarray	− 2.21
*AOC2*	Amine Oxidase, Copper Containing 2	Ouandaogo et al. 2011 [21]	Microarray	3.72
*AREG*	Amphiregulin	Feuerstein et al. 2007 [22]	RT-qPCR	5.4
*BDNF*	Brain Derived Neurotrophic Factor	Anderson et al. 2009 [23]	RT-qPCR	2.68
*BMP2*	Bone Morphogenetic Protein 2	Devjak et al. 2012 [19]	RNAseq	2.46
*BUB1*	BUB1 Mitotic Checkpoint Serine/Threonine Kinase	Devjak et al. 2012 [19]	RNAseq	− 4.28
		Feuerstein et al. 2012 [24]	Microarray	
*C10orf10*	aka. *DEPP1*, Autophagy Regulator	Devjak et al. 2012 [19]	RNAseq	2.99
*CCDC99*	aka. *SPDL1*, Spindle Apparatus Coiled-Coil Protein 1	Devjak et al. 2012 [19]	RNAseq	− 3.46
*CDH3*	Cadherin 3	Devjak et al. 2012 [19]	RNAseq	− 14.26
*COX2*	aka. *PTGS2*, Prostaglandin-Endoperoxide Synthase 2	Feuerstein et al. 2007 [22]	RT-qPCR	4.00
		Anderson et al. 2009 [23]	RT-qPCR	
		Wathlet et al. 2011 [25]	RT-qPCR	
		Yerushalmi et al. 2014 [20]	RNAseq	
*CRHBP*	Corticotropin Releasing Hormone Binding Protein	Devjak et al. 2012 [19]	RNAseq	− 5.41
*DHCR24*	24-Dehydrocholesterol Reductase	Yerushalmi et al. 2014 [20]	RNAseq	2.29
*DSE*	Dermatan Sulfate Epimerase	Devjak et al. 2012 [19]	RNAseq	− 2.36
*F2RL1*	F2R Like Trypsin Receptor 1	Ouandaogo et al. 2011 [21]	Microarray	− 3.06
*FSHR*	Follicle Stimulating Hormone Receptor	Yerushalmi et al. 2014 [20]	RNAseq	− 8.13
*GABRA5*	Gamma-Aminobutyric Acid Type A Receptor Alpha5 Subunit	Devjak et al. 2012 [19]	RNAseq	− 3.93
*GLRA2*	Glycine Receptor Alpha 2	Devjak et al. 2012 [19]	RNAseq	− 28.47
*GPX*	Glutathione Peroxidase 3	Yerushalmi et al. 2014 [20]	RNAseq	− 3.56
*GREM1*	Gremlin 1, DAN Family BMP Antagonist	Anderson et al. 2009 [23]	RT-qPCR	− 2.03
		Yerushalmi et al. 2014 [20]	RNAseq	
*HSD11B1*	Hydroxysteroid 11-Beta Dehydrogenase 1	Devjak et al. 2012 [19]	RNAseq	2.95
*ID2*	Inhibitor of DNA Binding 2	Ouandaogo et al. 2011 [21]	Microarray	3.53
*ID3*	Inhibitor of DNA Binding 3	Devjak et al. 2012 [19]	RNAseq	− 4.9
*ITGB3*	Integrin Subunit Beta 3	Devjak et al. 2012 [19]	RNAseq	− 4.05
*ITPKA*	Inositol-Trisphosphate 3-Kinase A	Wathlet et al. 2011 [25]	RT-qPCR	2.49
*LHCGR*	Luteinizing Hormone/Choriogonadotropin Receptor	Yerushalmi et al. 2014 [20]	RNAseq	3.72
*MAOB*	Monoamine Oxidase B	Devjak et al. 2012 [19]	RNAseq	− 2.38
*MGP*	Matrix Gla Protein	Devjak et al. 2012 [19]	RNAseq	− 8.01
*NDP*	Norrin Cystine Knot Growth Factor	Devjak et al. 2012 [19]	RNAseq	− 2.4
*NID2*	Nidogen 2	Devjak et al. 2012 [19]	RNAseq	5.46
*NKAIN1*	Sodium/Potassium Transporting ATPase Interacting 1	Devjak et al. 2012 [19]	RNAseq	4.38
*NOS2*	Nitric Oxide Synthase 2	Yerushalmi et al. 2014 [20]	RNAseq	− 2.48
*PALLD*	Palladin, Cytoskeletal Associated Protein	Devjak et al. 2012 [19]	RNAseq	− 4.13
*PTX3*	Pentraxin 3	Zhang et al. 2005 [26]	Microarray	3.08
		Anderson et al. 2009 [23]	RT-qPCR	
*SERPINE2*	Serpin Family E Member 2	Feuerstein et al. 2012 [24]	Microarray	− 4.31
		Yerushalmi et al. 2014 [20]	RNAseq	
*SFRP4*	Secreted Frizzled Related Protein 4	Devjak et al. 2012 [19]	RNAseq	− 20.39
		Feuerstein et al. 2012 [24]	Microarray	
		Yerushalmi et al. 2014 [20]	RNAseq	
*SPOCK2*	SPARC (Osteonectin), Cwcv And Kazal Like Domains Proteoglycan 2	Devjak et al. 2012 [19]	RNAseq	2.88
		Feuerstein et al. 2012 [24]	Microarray	
*STAR*	Steroidogenic Acute Regulatory Protein	Feuerstein et al. 2007 [22]	RT-qPCR	2.67
		Yerushalmi et al. 2014 [20]	RNAseq	
*TLL2*	Tolloid Like 2	Yerushalmi et al. 2014 [20]	RNAseq	− 3.17
*TNFSF4*	TNF Superfamily Member 4	Devjak et al. 2012 [19]	RNAseq	− 4.01
*TSPAN7*	Tetraspanin 7	Devjak et al. 2012 [19]	RNAseq	− 3.3

Correct Table 3:

**Table 3:** Potential oocyte maturation biomarkers

**Gene ID**	**Description**	**Previous Study**	**Method of Detection**	**Fold Change in this study**
*ADAMTS1*	ADAM Metallopeptidase with Thrombospondin Type 1 Motif 1	Devjak et al. 2012 [20]	RNAseq	2.27
		Yerushalmi et al. 2014 [21]	RNAseq	
*ANK2*	Ankyrin 2	Devjak et al. 2012 [20]	RNAseq	-3.13
*ANKRD57*	aka. *SOWAHC*, Sosondowah Ankyrin Repeat Domain Family Member C	Ouandaogo et al. 2011 [22]	Microarray	-2.21
*AOC2*	Amine Oxidase, Copper Containing 2	Ouandaogo et al. 2011 [22]	Microarray	3.72
*AREG*	Amphiregulin	Feuerstein et al. 2007 [23]	RT-qPCR	5.4
*BDNF*	Brain Derived Neurotrophic Factor	Anderson et al. 2009 [24]	RT-qPCR	2.68
*BMP2*	Bone Morphogenetic Protein 2	Devjak et al. 2012 [20]	RNAseq	2.46
*BUB1*	BUB1 Mitotic Checkpoint Serine/Threonine Kinase	Devjak et al. 2012 [20]	RNAseq	-4.28
		Feuerstein et al. 2012 [25]	Microarray	
*C10orf10*	aka. *DEPP1*, Autophagy Regulator	Devjak et al. 2012 [20]	RNAseq	2.99
*CCDC99*	aka. *SPDL1*, Spindle Apparatus Coiled-Coil Protein 1	Devjak et al. 2012 [20]	RNAseq	-3.46
*CDH3*	Cadherin 3	Devjak et al. 2012 [20]	RNAseq	-14.26
*COX2*	aka. *PTGS2*, Prostaglandin-Endoperoxide Synthase 2	Feuerstein et al. 2007 [23]	RT-qPCR	4.00
		Anderson et al. 2009 [24]	RT-qPCR	
		Wathlet et al. 2011 [26]	RT-qPCR	
		Yerushalmi et al. 2014 [21]	RNAseq	
*CRHBP*	Corticotropin Releasing Hormone Binding Protein	Devjak et al. 2012 [20]	RNAseq	-5.41
*DHCR24*	24-Dehydrocholesterol Reductase	Yerushalmi et al. 2014 [21]	RNAseq	2.29
*DSE*	Dermatan Sulfate Epimerase	Devjak et al. 2012 [20]	RNAseq	-2.36
*F2RL1*	F2R Like Trypsin Receptor 1	Ouandaogo et al. 2011 [22]	Microarray	-3.06
*FSHR*	Follicle Stimulating Hormone Receptor	Yerushalmi et al. 2014 [21]	RNAseq	-8.13
*GABRA5*	Gamma-Aminobutyric Acid Type A Receptor Alpha5 Subunit	Devjak et al. 2012 [20]	RNAseq	-3.93
*GLRA2*	Glycine Receptor Alpha 2	Devjak et al. 2012 [20]	RNAseq	-28.47
*GPX*	Glutathione Peroxidase 3	Yerushalmi et al. 2014 [21]	RNAseq	-3.56
*GREM1*	Gremlin 1, DAN Family BMP Antagonist	Anderson et al. 2009 [24]	RT-qPCR	-2.03
		Yerushalmi et al. 2014 [21]	RNAseq	
*HSD11B1*	Hydroxysteroid 11-Beta Dehydrogenase 1	Devjak et al. 2012 [20]	RNAseq	2.95
*ID2*	Inhibitor of DNA Binding 2	Ouandaogo et al. 2011 [22]	Microarray	3.53
*ID3*	Inhibitor of DNA Binding 3	Devjak et al. 2012 [20]	RNAseq	-4.9
*ITGB3*	Integrin Subunit Beta 3	Devjak et al. 2012 [20]	RNAseq	-4.05
*ITPKA*	Inositol-Trisphosphate 3-Kinase A	Wathlet et al. 2011 [26]	RT-qPCR	2.49
*LHCGR*	Luteinizing Hormone/Choriogonadotropin Receptor	Yerushalmi et al. 2014 [21]	RNAseq	3.72
*MAOB*	Monoamine Oxidase B	Devjak et al. 2012 [20]	RNAseq	-2.38
*MGP*	Matrix Gla Protein	Devjak et al. 2012 [20]	RNAseq	-8.01
*NDP*	Norrin Cystine Knot Growth Factor	Devjak et al. 2012 [20]	RNAseq	-2.4
*NID2*	Nidogen 2	Devjak et al. 2012 [20]	RNAseq	5.46
*NKAIN1*	Sodium/Potassium Transporting ATPase Interacting 1	Devjak et al. 2012 [20]	RNAseq	4.38
*NOS2*	Nitric Oxide Synthase 2	Yerushalmi et al. 2014 [21]	RNAseq	-2.48
*PALLD*	Palladin, Cytoskeletal Associated Protein	Devjak et al. 2012 [20]	RNAseq	-4.13
*PTX3*	Pentraxin 3	Zhang et al. 2005 [27]	Microarray	3.08
		Anderson et al. 2009 [24]	RT-qPCR	
*SERPINE2*	Serpin Family E Member 2	Feuerstein et al. 2012 [25]	Microarray	-4.31
		Yerushalmi et al. 2014 [21]	RNAseq	
*SFRP4*	Secreted Frizzled Related Protein 4	Devjak et al. 2012 [20]	RNAseq	-20.39
		Feuerstein et al. 2012 [25]	Microarray	
		Yerushalmi et al. 2014 [21]	RNAseq	
*SPOCK2*	SPARC (Osteonectin), Cwcv And Kazal Like Domains Proteoglycan 2	Devjak et al. 2012 [20]	RNAseq	2.88
		Feuerstein et al. 2012 [25]	Microarray	
*STAR*	Steroidogenic Acute Regulatory Protein	Feuerstein et al. 2007 [23]	RT-qPCR	2.67
		Yerushalmi et al. 2014 [21]	RNAseq	
*TLL2*	Tolloid Like 2	Yerushalmi et al. 2014 [21]	RNAseq	-3.17
*TNFSF4*	TNF Superfamily Member 4	Devjak et al. 2012 [20]	RNAseq	-4.01
*TSPAN7*	Tetraspanin 7	Devjak et al. 2012 [20]	RNAseq	-3.3

2. Reference citations in main body text were incorrectly cited. Correct citations were listed in the below table.

**Table:** 

**Incorrect**	**Correct**
**Novel findings from this study enhance available literature exploring processes that lead to synchronized oocyte maturity section:**	
Forty-five genes previously correlated with oocyte maturation were not differentially expressed in the current study (Supplemental Table S4) [27]. Three thousand five hundred and fifty-four genes	Forty-five genes previously correlated with oocyte maturation were not differentially expressed in the current study (Supplemental Table S4) [19]. Three thousand five hundred and fifty-four genes
**Discussion section:**	
Previous human oocyte maturation studies analyzed COCs from in-vitro maturation cycles [16, 29–33]	Previous human oocyte maturation studies analyzed COCs from in-vitro maturation cycles [16, 21, 29–32]
In this study, several factors and their regulators involved in nuclear maturation and cell cycle control were differentially expressed between cumulus cells encapsulating oocytes of different maturity, reiterating findings from previous studies [19, 20, 29, 34]. These include cell cycle regulators (BIRC5, BUB1, BUB1B, CCNA2, CCNB, CDK1, FBXO5 MAD2L1, and PTTG1) and components of the centromere (CENPA, CENPE, and CENPH) [29]. In our MII-CC cohort we observed downregulation of MCM2–7, which form the hexameric pre-replication protein complex. This complex is involved in initiating replication forks and recruiting other DNA replication related proteins. We also observed downregulation of TOP2A, which relaxes supercoiled and circular DNA molecules. Reinforcing available literature that states that while crucial at the MI stage for chromatin remodeling [21, 22], its activity decreases in mature oocytes [23]	In this study, several factors and their regulators involved in nuclear maturation and cell cycle control were differentially expressed between cumulus cells encapsulating oocytes of different maturity, reiterating findings from previous studies [21, 22, 33, 34]. These include cell cycle regulators (BIRC5, BUB1, BUB1B, CCNA2, CCNB, CDK1, FBXO5 MAD2L1, and PTTG1) and components of the centromere (CENPA, CENPE, and CENPH) [21].]. In our MII-CC cohort we observed downregulation of MCM2–7, which form the hexameric pre-replication protein complex. This complex is involved in initiating replication forks and recruiting other DNA replication related proteins. We also observed downregulation of TOP2A, which relaxes supercoiled and circular DNA molecules. Reinforcing available literature that states that while crucial at the MI stage for chromatin remodeling [35, 36], its activity decreases in mature oocytes [37]
Apoptosis was also attenuated in the MII-CC cohort, further supporting decreased cell turnover with advanced maturity. Related pathways including Wnt pathway and Akt-pathway were affected, as demonstrated by downregulation of SFRP4, a potent inhibitor of Wnt signaling [24], and upregulation of OSMR, an activator of Akt-mediated proliferation [25]. These findings corroborate previous literature reporting downregulation of SFRP4 during oocyte maturation [26, 35, 36], and upregulation of OSMR in bovine preovulatory follicles posttriggering by gonadotropins [37]	Apoptosis was also attenuated in the MII-CC cohort, further supporting decreased cell turnover with advanced maturity. Related pathways including Wnt pathway and Akt-pathway were affected, as demonstrated by downregulation of SFRP4, a potent inhibitor of Wnt signaling [38], and upregulation of OSMR, an activator of Akt-mediated proliferation [39]. These findings corroborate previous literature reporting downregulation of SFRP4 during oocyte maturation [20, 40, 41], and upregulation of OSMR in bovine preovulatory follicles posttriggering by gonadotropins [42]
Extracellular matrix remodeling was also altered between the two maturity cohorts, as evident by members of the matrix metalloproteinases (MMP) family and their inducers (MMP11 and SPARC1L). Again, this supports previous literature showing significant decrease of MMP11 in granulosa cells following hCG administration [38]. This effect is further demonstrated by increased expression of TNC, NID2, and SPOCK2—all ECM proteins and MMP substrates [26, 39–41]. Notably, well characterized ECM remodeling enzymes, ADAMTS1 and SERPINE2, were also differentially expressed, aligning with previous studies [42, 43]. Both play critical roles in follicular remodeling during follicular growth and rupture [44], by metabolizing Versican and Hyaluronan which lead to cumulus cell matrix expansion and attenuation [45]	Extracellular matrix remodeling was also altered between the two maturity cohorts, as evident by members of the matrix metalloproteinases (MMP) family and their inducers (MMP11 and SPARC1L). Again, this supports previous literature showing significant decrease of MMP11 in granulosa cells following hCG administration [43]. This effect is further demonstrated by increased expression of TNC, NID2, and SPOCK2—all ECM proteins and MMP substrates [20, 44–46]. Notably, well characterized ECM remodeling enzymes, ADAMTS1 and SERPINE2, were also differentially expressed, aligning with previous studies [47, 48]. Both play critical roles in follicular remodeling during follicular growth and rupture [49], by metabolizing Versican and Hyaluronan which lead to cumulus cell matrix expansion and attenuation [50]
Another key process enhanced in follicular niche maturation is inflammation, which is crucial for ovulation. Upon gonadotropin stimulation, the follicle wall is weakened, thereby facilitating its eventual rupture [46]. In our MII-CC cohort, we observed marked upregulation of genes associated with inflammation, including members of the Interleukin and TGF-beta families. Among the genes upregulated in our MII-CC cohort were IL18R1 which promotes cumulus cell expansion [47], and TGFBR3 which promotes cellular differentiation, migration, adhesion and extracellular matrix production [48, 49]. IL6ST which is part of the cytokine receptor complex (gp130) was also upregulated in the MII-CC cohort, consistent with previous studies in non-human primates and equine models [50, 51]	Another key process enhanced in follicular niche maturation is inflammation, which is crucial for ovulation. Upon gonadotropin stimulation, the follicle wall is weakened, thereby facilitating its eventual rupture [51]. In our MII-CC cohort, we observed marked upregulation of genes associated with inflammation, including members of the Interleukin and TGF-beta families. Among the genes upregulated in our MII-CC cohort were IL18R1 which promotes cumulus cell expansion [52], and TGFBR3 which promotes cellular differentiation, migration, adhesion and extracellular matrix production [53, 54]. IL6ST which is part of the cytokine receptor complex (gp130) was also upregulated in the MII-CC cohort, consistent with previous studies in non-human primates and equine models [55, 56]
Key players that emerged in our cohort as being significant for cumulus cells to facilitate oocyte maturation are AREG, EREG, PTGS2, and STAR. Two factors at the heart of this complex process are AREG and EREG, which have been shown to mediate the LH signal driving cumulus expansion and oocyte maturation [19, 32, 52]. They also activate the EGF receptor (EGFR) which in turn releases matrix metalloproteinases (MMPs) and promotes cumulus expansion [52, 53]. Furthermore, in conjunction with progesterone, AREG and EREG enhance PTGS2 (also upregulated in our MII-CC cohort) via EGF to increase prostaglandin production and maintenance of chromosomal spindles [33, 54–56]. In addition, AREG mediates hCG-induced STAR expression (also upregulated in our MII-CC cohort), which plays a key role in steroid and progesterone production in human granulosa cells [57], and is a potential predictive biomarker for nuclear maturation [58] and oocyte quality [33]. It is important to note, that despite being well defined as key in ovarian maturation [32, 52, 59], EREG has not been found to be differentially expressed in previous genomic signature studies addressing this question. This further highlights the importance of our study design in better refining the pathophysiology of oocyte maturation	Key players that emerged in our cohort as being significant for cumulus cells to facilitate oocyte maturation are AREG, EREG, PTGS2, and STAR. Two factors at the heart of this complex process are AREG and EREG, which have been shown to mediate the LH signal driving cumulus expansion and oocyte maturation [31, 33, 57]. They also activate the EGF receptor (EGFR) which in turn releases matrix metalloproteinases (MMPs) and promotes cumulus expansion [57, 58]. Furthermore, in conjunction with progesterone, AREG and EREG enhance PTGS2 (also upregulated in our MII-CC cohort) via EGF to increase prostaglandin production and maintenance of chromosomal spindles [32, 59–61]. In addition, AREG mediates hCG-induced STAR expression (also upregulated in our MII-CC cohort), which plays a key role in steroid and progesterone production in human granulosa cells [62], and is a potential predictive biomarker for nuclear maturation [23] and oocyte quality [32]. It is important to note, that despite being well defined as key in ovarian maturation [31, 57, 63], EREG has not been found to be differentially expressed in previous genomic signature studies addressing this question. This further highlights the importance of our study design in better refining the pathophysiology of oocyte maturation
IL1 (both alpha and beta subunits), which stimulates steroidogenesis, was upregulated in the MII-CC cohort with a concurrent decreased expression of FSHR in the same cohort, substantiating what was previously observed in rodents and humans [60, 61]. BDNF, which modulates granulosa cell function via FSHR-coupled signaling pathway, to affect aromatase-mediated steroidogenesis, was also downregulated in our MII-CC cohort [62]	IL1 (both alpha and beta subunits), which stimulates steroidogenesis, was upregulated in the MII-CC cohort with a concurrent decreased expression of FSHR in the same cohort, substantiating what was previously observed in rodents and humans [64, 65]. BDNF, which modulates granulosa cell function via FSHR-coupled signaling pathway, to affect aromatase-mediated steroidogenesis, was also downregulated in our MII-CC cohort [66]
HSD11B1, the enzyme responsible for cortisone production, an essential substrate for steroid hormone synthesis, was upregulated in our MII-CC cohort. A companion enzyme, HSD17B1, catalyzes the last step in estrogen metabolism converting E1 of low estrogenic activity to E2 of high activity using cortisone as a substrate [63]. HSD17B1 has not been captured in previous human studies, but was downregulated in our MII-CC cohort, consistent with the results seen in a previous bovine study [64], and further highlighting the advantage of our study design	HSD11B1, the enzyme responsible for cortisone production, an essential substrate for steroid hormone synthesis, was upregulated in our MII-CC cohort. A companion enzyme, HSD17B1, catalyzes the last step in estrogen metabolism converting E1 of low estrogenic activity to E2 of high activity using cortisone as a substrate [67]. HSD17B1 has not been captured in previous human studies, but was downregulated in our MII-CC cohort, consistent with the results seen in a previous bovine study [68], and further highlighting the advantage of our study design
Overall, apoptosis was enriched in downregulated genes. Interestingly, several major players in the regulation of apoptosis, including BIRC5, TP53, HMGB1, HMGB2, and SFRP4 are also known to be regulated by LH and/or FSH [24, 35, 65–67]	Overall, apoptosis was enriched in downregulated genes. Interestingly, several major players in the regulation of apoptosis, including BIRC5, TP53, HMGB1, HMGB2, and SFRP4 are also known to be regulated by LH and/or FSH [38, 40, 69–71]
Overall, biosynthesis was enriched in upregulated genes among the MII-CC cohort. Notably, several members of the CYP family, which were upregulated, and are involved in the biosynthesis of estrogen and androgens, are known to be regulated by LH and/or FSH [68–70]	Overall, biosynthesis was enriched in upregulated genes among the MII-CC cohort. Notably, several members of the CYP family, which were upregulated, and are involved in the biosynthesis of estrogen and androgens, are known to be regulated by LH and/or FSH [72–74]
Finally, we show that PDE3A, known to improve nuclear-cytoplasmic synchrony [71], is significantly upregulated in our MII-CC cohort. While this gene has not been studied in cumulus cells in the context of oocyte maturation in humans, it has been shown that an increase in oocyte PDE3A activity causes delayed spontaneous meiotic maturation, coupled with extended gap junctional communication between the CC and the oocyte. Such a delay has a positive effect on oocyte cytoplasmic maturation, thereby improving oocyte developmental potential [72]. The fact that upregulation of this gene was captured by our study design speaks once again to the strength of our study and to what it adds to current literature	Finally, we show that PDE3A, known to improve nuclear-cytoplasmic synchrony [75], is significantly upregulated in our MII-CC cohort. While this gene has not been studied in cumulus cells in the context of oocyte maturation in humans, it has been shown that an increase in oocyte PDE3A activity causes delayed spontaneous meiotic maturation, coupled with extended gap junctional communication between the CC and the oocyte. Such a delay has a positive effect on oocyte cytoplasmic maturation, thereby improving oocyte developmental potential [76]. The fact that upregulation of this gene was captured by our study design speaks once again to the strength of our study and to what it adds to current literature
Methodological strengths of this study include (i) a sibling COC design allowing to minimize the biologic variability between cohorts, (ii) exploring transcriptomic dynamics in cumulus cells, which are considered valuable non-invasive markers for oocyte quality [73–75], and (iii) performing next generation sequencing (NGS), which is the most unbiased approach currently available for exploring transcriptomic signatures	Methodological strengths of this study include (i) a sibling COC design allowing to minimize the biologic variability between cohorts, (ii) exploring transcriptomic dynamics in cumulus cells, which are considered valuable non-invasive markers for oocyte quality [77–79], and (iii) performing next generation sequencing (NGS), which is the most unbiased approach currently available for exploring transcriptomic signatures
**Differential expression section:**	
Raw trimmed reads were aligned to Human Genome Assembly 38 (hg38) using STAR (v2.5.3a) [77] and quantified to RefSeq (Release 84). Low expressed transcripts were excluded (maximum counts < 10) and differential expression (DE) was conducted on the remaining counts using DESeq2 (v3.5) [78]	Raw trimmed reads were aligned to Human Genome Assembly 38 (hg38) using STAR (v2.5.3a) [81] and quantified to RefSeq (Release 84). Low expressed transcripts were excluded (maximum counts < 10) and differential expression (DE) was conducted on the remaining counts using DESeq2 (v3.5) [82]
**Pathway analysis section:**	
The resulting pathway list was cross referenced with a custom gene set created and supported by the Bader Lab (University of Toronto) which is comprised of all GO database, KEGG, and Reactome gene sets (v2018-12–01) (http://download.baderlab.org/EM_Genesets/) [79]	The resulting pathway list was cross referenced with a custom gene set created and supported by the Bader Lab (University of Toronto) which is comprised of all GO database, KEGG, and Reactome gene sets (v2018-12–01) (http://download.baderlab.org/EM_Genesets/) [83]
To further explore the impact FSH and/or LH may have on the transcriptome, we identified all differentially expressed genes that are known to be regulated by LH, FSH or both [80] and performed GSEA and LEA as described previously	To further explore the impact FSH and/or LH may have on the transcriptome, we identified all differentially expressed genes that are known to be regulated by LH, FSH or both [84] and performed GSEA and LEA as described previously
Relative fold change (ΔΔCt) was employed to quantify gene expression [81]	Relative fold change (ΔΔCt) was employed to quantify gene expression [85]
**Gene annotation and literature search section:**	
Differentially expressed genes were further reviewed in depth using the Ovarian Kaleidoscope Database [80] and GeneCards Human Gene databases (http://www.genecards.org/), to correlate our bioinformatic findings with hallmark physiological and pathological processes in the ovary	Differentially expressed genes were further reviewed in depth using the Ovarian Kaleidoscope Database [84] and GeneCards Human Gene databases (http://www.genecards.org/), to correlate our bioinformatic findings with hallmark physiological and pathological processes in the ovary

3. References Anderson et al. 2009; Feuerstein et al. 2012; Wathlet et al. 2011; Zhang et al. 2005 were not listed in the reference list. Below are the reference details:
  a Anderson RA, Sciorio R, Kinnell H, Bayne RAL, Thong KJ, Sousa PAd, et al. Cumulus gene expression as a predictor of human oocyte fertilisation, embryo development and competence to establish a pregnancy. REPRODUCTION. 2009;138(4):629–37.
  b Feuerstein P, Puard V, Chevalier C, Teusan R, Cadoret V, Guerif F, et al. Genomic Assessment of Human Cumulus Cell Marker Genes as Predictors of Oocyte Developmental Competence: Impact of Various Experimental Factors. PLoS ONE. 2012;7(7):e40449.
  c Wathlet S, Adriaenssens T, Segers I, Verheyen G, Van de Velde H, Coucke W, et al. Cumulus cell gene expression predicts better cleavage-stage embryo or blastocyst development and pregnancy for ICSI patients. Human Reproduction. 2011;26(5):1035–51.
  d Zhang X, Jafari N, Barnes RB, Confino E, Milad M, Kazer RR. Studies of gene expression in human cumulus cells indicate pentraxin 3 as a possible marker for oocyte quality. Fertility and Sterility. 2005;83(4):1169–79.
4. The reference list was incorrectly numbered. Below is the correct reference list.

1. Eppig JJ. Oocyte control of ovarian follicular development and function in mammals. Reproduction. 2001;122(6):829–38.

2. Ackert CL, Gittens JE, O'Brien MJ, Eppig JJ, Kidder GM. Intercellular communication via connexin43 gap junctions is required for ovarian folliculogenesis in the mouse. Dev Biol. 2001;233(2):258–70.

3. Matzuk MM, Burns KH, Viveiros MM, Eppig JJ. Intercellular communication in the mammalian ovary: oocytes carry the conversation. Science. 2002; 296(5576):2178–80.

4. Gilchrist RB, Lane M, Thompson JG. Oocyte-secreted factors: regulators of cumulus cell function and oocyte quality. Hum Reprod Update. 2008;14(2): 159–77.

5. McKenzie LJ, Pangas SA, Carson SA, Kovanci E, Cisneros P, Buster JE, et al. Human cumulus granulosa cell gene expression: a predictor of fertilization and embryo selection in women undergoing IVF. Hum Reprod. 2004;19(12): 2869–74.

6. van Montfoort APA, Geraedts JPM, Dumoulin JCM, Stassen APM, Evers JLH, Ayoubi TAY. Differential gene expression in cumulus cells as a prognostic indicator of embryo viability: a microarray analysis. Mol Hum Reprod. 2008; 14(3):157–68.

7. Su YQ, Sugiura K, Woo Y, Wigglesworth K, Kamdar S, Affourtit J, et al. Selective degradation of transcripts during meiotic maturation of mouse oocytes. Dev Biol. 2007;302(1):104–17.

8. Eichenlaub-Ritter U, Vogt E, Yin H, Gosden R. Spindles, mitochondria and redox potential in ageing oocytes. Reprod BioMed Online. 2004;8(1):45–58.

9. Eppig JJ. Reproduction: oocytes call, Granulosa Cells Connect. Curr Biol. 2018;28(8):R354–R6.

10. Bachvarova R. Gene expression during oogenesis and oocyte development in mammals. Dev Biol (N Y 1985). 1985;1:453–524.

11. Braude P, Bolton V, Moore S. Human gene expression first occurs between the four- and eight-cell stages of preimplantation development. Nature. 1988;332(6163):459–61.

12. De La Fuente R, Viveiros MM, Burns KH, Adashi EY, Matzuk MM, Eppig JJ. Major chromatin remodeling in the germinal vesicle (GV) of mammalian oocytes is dispensable for global transcriptional silencing but required for centromeric heterochromatin function. Dev Biol. 2004;275(2):447–58.

13. Macaulay AD, Gilbert I, Scantland S, Fournier E, Ashkar F, Bastien A, et al. Cumulus Cell Transcripts Transit to the Bovine Oocyte in Preparation for Maturation. Biol Reprod. 2016;94(1):16.

14. Macaulay AD, Gilbert I, Caballero J, Barreto R, Fournier E, Tossou P, et al. The gametic synapse: RNA transfer to the bovine oocyte. Biol Reprod. 2014;91(4):90.

15. Baena V, Terasaki M. Three-dimensional organization of transzonal projections and other cytoplasmic extensions in the mouse ovarian follicle. Sci Rep. 2019;9(1):1262.

16. Wells D, Patrizio P. Gene expression profiling of human oocytes at different maturational stages and after in vitro maturation. Am J Obstet Gynecol. 2008;198(4):455 e1–9 discussion e9–11.

17. Ching T, Huang S, Garmire LX. Power analysis and sample size estimation for RNA-Seq differential expression. RNA (New York). 2014;20(11):1684–96.

18. Busby MA, Stewart C, Miller CA, Grzeda KR, Marth GT. Scotty: a web tool for designing RNA-Seq experiments to measure differential gene expression. Bioinformatics. 2013;29(5):656–7.

19. Iager AE, Kocabas AM, Otu HH, Ruppel P, Langerveld A, Schnarr P, et al. Identification of a novel gene set in human cumulus cells predictive of an oocyte's pregnancy potential. Fertil Steril. 2013;99(3):745–52.e6.

20. Devjak R, Fon Tacer K, Juvan P, Virant Klun I, Rozman D, Vrtačnik BE. Cumulus cells gene expression profiling in terms of oocyte maturity in controlled ovarian Hyperstimulation using GnRH agonist or GnRH antagonist. PLoS One. 2012;7(10):e47106.

21. Yerushalmi GM, Salmon-Divon M, Yung Y, Maman E, Kedem A, Ophir L, et al. Characterization of the human cumulus cell transcriptome during final follicular maturation and ovulation. Mol Hum Reprod. 2014;20(8):719–35.

22. Ouandaogo ZG, Haouzi D, Assou S, Dechaud H, Kadoch IJ, De Vos J, et al. Human cumulus cells molecular signature in relation to oocyte nuclear maturity stage. PloS one. 2011;6(11):e27179-e.

23. Feuerstein P, Cadoret V, Dalbies-Tran R, Guerif F, Bidault R, Royere D. Gene expression in human cumulus cells: one approach to oocyte competence. Hum Reprod. 2007;22(12):3069–77.

24. Anderson RA, Sciorio R, Kinnell H, Bayne RAL, Thong KJ, Sousa PAd, et al. Cumulus gene expression as a predictor of human oocyte fertilisation, embryo development and competence to establish a pregnancy. REPRODUCTION. 2009;138(4):629–37.

25. Feuerstein P, Puard V, Chevalier C, Teusan R, Cadoret V, Guerif F, et al. Genomic Assessment of Human Cumulus Cell Marker Genes as Predictors of Oocyte Developmental Competence: Impact of Various Experimental Factors. PLoS ONE. 2012;7(7):e40449.

26. Wathlet S, Adriaenssens T, Segers I, Verheyen G, Van de Velde H, Coucke W, et al. Cumulus cell gene expression predicts better cleavage-stage embryo or blastocyst development and pregnancy for ICSI patients. Human Reproduction. 2011;26(5):1035–51.

27. Zhang X, Jafari N, Barnes RB, Confino E, Milad M, Kazer RR. Studies of gene expression in human cumulus cells indicate pentraxin 3 as a possible marker for oocyte quality. Fertility and Sterility. 2005;83(4):1169–79.

28. Subramanian A, Tamayo P, Mootha VK, Mukherjee S, Ebert BL, Gillette MA, et al. Gene set enrichment analysis: a knowledge-based approach for interpreting genome-wide expression profiles. Proc Natl Acad Sci. 2005; 102(43):15,545–50.

29. Molinari E, Bar H, Pyle AM, Patrizio P. Transcriptome analysis of human cumulus cells reveals hypoxia as the main determinant of follicular senescence. Mol Hum Reprod. 2016;22(8):866–76.

30. Hannah MB, Kylie RD, Melanie S-M, Robert BG, Jeremy GT, Darryl LR. Failure to launch: aberrant cumulus gene expression during oocyte in vitro maturation. Reproduction. 2017;153(3):R109–R20.

31. Ben-Ami I, Komsky A, Bern O, Kasterstein E, Komarovsky D, Ron-El R. In vitro maturation of human germinal vesicle-stage oocytes: role of epidermal growth factor-like growth factors in the culture medium. Hum Reprod. 2011;26(1):76–81.

32. Dhali A, Javvaji PK, Kolte AP, Francis JR, Roy SC, Sejian V. Temporal expression of cumulus cell marker genes during in vitro maturation and oocyte developmental competence. J Assist Reprod Genet. 2017;34(11): 1493–500.

33. Ouandaogo ZG, Frydman N, Hesters L, Assou S, Haouzi D, Dechaud H, et al. Differences in transcriptomic profiles of human cumulus cells isolated from oocytes at GV, MI and MII stages after in vivo and in vitro oocyte maturation. Hum Reprod. 2012;27(8):2438–47.

34. Wei L, Liang X-W, Zhang Q-H, Li M, Yuan J, Li S, et al. BubR1 is a spindle assembly checkpoint protein regulating meiotic cell cycle progression of mouse oocyte. Cell Cycle. 2010;9(6):1112–21.

35. Li X-M, Yu C, Wang Z-W, Zhang Y-L, Liu X-M, Zhou D, et al. DNA Topoisomerase II Is Dispensable for Oocyte Meiotic Resumption but Is Essential for Meiotic Chromosome Condensation and Separation in Mice1. Biology of Reproduction. 2013;89(5):1–11.

36. Assou S, Cerecedo D, Tondeur S, Pantesco V, Hovatta O, Klein B, et al. A gene expression signature shared by human mature oocytes and embryonic stem cells. BMC Genomics. 2009;10:10.

37. Yerushalmi GM, Salmon-Divon M, Ophir L, Yung Y, Baum M, Coticchio G, et al. Characterization of the miRNA regulators of the human ovulatory cascade. Sci Rep. 2018;8(1):15,605.

38. M Drake J, R Friis R, Dharmarajan A. The role of sFRP4, a secreted frizzledrelated protein, in ovulation 2003. 389–97 p.

39. Abir R, Ao A, Jin S, Barnett M, Van den Hurk R, Freimann S, et al. Immunocytochemical detection and reverse transcription polymerase chain reaction expression of oncostatin M (OSM) and its receptor (OSM-Rβ) in human fetal and adult ovaries. Fertil Steril. 2005;83(4):1188–96.

40. Maman E, Yung Y, Cohen B, Konopnicki S, Dal Canto M, Fadini R, et al. Expression and regulation of sFRP family members in human granulosa cells. Mol Hum Reprod. 2011;17(7):399–404.

41. Xin H, Cuifang H, Xiaofang S, Xiaoyan L, Yinghua S, Yuhua Z, et al. Differences in the transcriptional profiles of human cumulus cells isolated from MI and MII oocytes of patients with polycystic ovary syndrome. Reproduction. 2013;145(6):597–608.

42. Martins KR, Haas CS, Ferst JG, Rovani MT, Goetten ALF, Duggavathi R, et al. Oncostatin M and its receptors mRNA regulation in bovine granulosa and luteal cells. Theriogenology. 2019;125:324–30.

43. McCord LA, Li F, Rosewell KL, Brännström M, Curry TE. Ovarian expression and regulation of the stromelysins during the periovulatory period in the human and the rat. Biol Reprod. 2011;86(3):78.

44. Yasuda K, Hagiwara E, Takeuchi A, Mukai C, Matsui C, Sakai A, et al. Changes in the distribution of Tenascin and Fibronectin in the mouse ovary during Folliculogenesis, atresia, Corpus Luteum formation and Luteolysis. Zool Sci. 2005;22(2):237–45 9.

45. Bagavandoss P. Temporal expression of tenascin-C and type I collagen in response to gonadotropins in the immature rat ovary. Acta Histochem. 2014;116(7):1125–33.

46. Hernandez-Gonzalez I, Gonzalez-Robayna I, Shimada M, Wayne CM, Ochsner SA, White L, et al. Gene expression profiles of cumulus cell oocyte complexes during ovulation reveal cumulus cells express neuronal and immune-related genes: does this expand their role in the ovulation process? Mol Endocrinol. 2006;20(6):1300–21.

47. Lu CH, Lee RK, Hwu YM, Lin MH, Yeh LY, Chen YJ, et al. Involvement of the serine protease inhibitor, SERPINE2, and the urokinase plasminogen activator in cumulus expansion and oocyte maturation. PLoS One. 2013;8(8):e74602.

48. Pan B, Li J. MicroRNA-21 up-regulates metalloprotease by down-regulating TIMP3 during cumulus cell-oocyte complex in vitro maturation. Mol Cell Endocrinol. 2018;477:29–38.

49. Sayasith K, Lussier J, Sirois J. Molecular characterization and transcriptional regulation of a disintegrin and metalloproteinase with thrombospondin motif 1 (ADAMTS1) in bovine preovulatory follicles. Endocrinology. 2013; 154(8):2857–69.

50. Young KA, Tumlinson B, Stouffer RL. ADAMTS-1/METH-1 and TIMP-3 expression in the primate corpus luteum: divergent patterns and stagedependent regulation during the natural menstrual cycle. Mol Hum Reprod. 2004;10(8):559–65.

51. Russell DL, Robker RL. Molecular mechanisms of ovulation: co-ordination through the cumulus complex. Hum Reprod Update. 2007;13(3):289–312.

52. Tsuji Y, Tamaoki TH, Hasegawa A, Kashiwamura S, Iemoto A, Ueda H, et al. Expression of interleukin-18 and its receptor in mouse ovary. Am J Reprod Immunol. 2001;46(5):349–57.

53. Wang S, Liu W, Pang X, Dai S, Liu G. The Mechanism of Melatonin and Its Receptor MT2 Involved in the Development of Bovine Granulosa Cells. International Journal of Molecular Sciences. 2018;19(7). https://www.mdpi.com/about/announcements/784.

54. Myers M, van den Driesche S, McNeilly AS, Duncan WC. Activin a reduces luteinisation of human luteinised granulosa cells and has opposing effects to human chorionic gonadotropin in vitro. J Endocrinol. 2008;199(2):201–12.

55. Murphy MJ, Halow NG, Royer PA, Hennebold JD. Leukemia inhibitory factor is necessary for ovulation in female rhesus macaques. Endocrinology. 2016; 157(11):4378–87.

56. Sessions-Bresnahan DR, Carnevale EM. Age-associated changes in granulosa cell transcript abundance in equine preovulatory follicles. Reprod Fertil Dev. 2015;27(6):906–13.

57. Park JY, Su YQ, Ariga M, Law E, Jin SL, Conti M. EGF-like growth factors as mediators of LH action in the ovulatory follicle. Science. 2004;303(5658): 682–4.

58. Light A, Hammes SR. LH-induced Steroidogenesis in the mouse ovary, but not testis, requires matrix metalloproteinase 2- and 9-mediated cleavage of Upregulated EGF receptor ligands. Biol Reprod. 2015;93(3):65.

59. Choi Y, Wilson K, Hannon PR, Rosewell KL, Brännström M, Akin JW, et al. Coordinated regulation among progesterone, prostaglandins, and EGF-like factors in human ovulatory follicles. J Clin Endocrinol Metab. 2017;102(6): 1971–82.

60. Nuttinck F, Gall L, Ruffini S, Laffont L, Clement L, Reinaud P, et al. PTGS2-related PGE2 affects oocyte MAPK phosphorylation and meiosis progression in cattle: late effects on early embryonic development. Biol Reprod. 2011; 84(6):1248–57.

61. Marei WF, Abayasekara DR, Wathes DC, Fouladi-Nashta AA. Role of PTGS2-generated PGE2 during gonadotrophin-induced bovine oocyte maturation and cumulus cell expansion. Reprod BioMed Online. 2014;28(3):388–400.

62. Fang L, Yu Y, Zhang R, He J, Sun YP. Amphiregulin mediates hCG-induced StAR expression and progesterone production in human granulosa cells. Sci Rep. 2016;6:24,917.

63. Ashkenazi H, Cao X, Motola S, Popliker M, Conti M, Tsafriri A. Epidermal growth factor family members: endogenous mediators of the ovulatory response. Endocrinology. 2005;146(1):77–84.

64. Calder MD, Caveney AN, Smith LC, Watson AJ. Responsiveness of bovine cumulus-oocyte-complexes (COC) to porcine and recombinant human FSH, and the effect of COC quality on gonadotropin receptor and Cx43 marker gene mRNAs during maturation in vitro. Reprod Biol Endocrinol. 2003;1:14.

65. Guzman L, Adriaenssens T, Ortega-Hrepich C, Albuz FK, Mateizel I, Devroey P, et al. Human antral follicles < 6 mm: a comparison between in vivo maturation and in vitro maturation in non-hCG primed cycles using cumulus cell gene expression. Mol Hum Reprod. 2013;19(1):7–16.

66. Xie M, Li M, Zhou J, Ding X, Shao Y, Jing J, et al. Brain-derived neurotrophic factor promotes human granulosa-like tumor cell steroidogenesis and proliferation by activating the FSH receptor-mediated signaling pathway. Sci Rep. 2017;7(1):180.

67. Järvensivu P, Saloniemi-Heinonen T, Awosanya M, Koskimies P, Saarinen N, Poutanen M. HSD17B1 expression enhances estrogen signaling stimulated by the low active estrone, evidenced by an estrogen responsive elementdriven reporter gene in vivo. Chem Biol Interact. 2015;234:126–34.

68. de Souza DK, Salles LP, Camargo R, Gulart LVM, Costa E, Silva S, de Lima BD, et al. Effects of PI3K and FSH on steroidogenesis, viability and embryo development of the cumulus-oocyte complex after in vitro culture. Zygote. 2018;26(1):50–61.

69. Leon PM, Campos VF, Kaefer C, Begnini KR, McBride AJ, Dellagostin OA, et al. Expression of apoptotic genes in immature and in vitro matured equine oocytes and cumulus cells. Zygote. 2013;21(3):279–85.

70. Kumazawa Y, Kawamura K, Sato T, Sato N, Konishi Y, Shimizu Y, et al. HCG up-regulates survivin mRNA in human granulosa cells. Mol Hum Reprod. 2005;11(3):161–6.

71. Ni XR, Sun ZJ, Hu GH, Wang RH. High concentration of insulin promotes apoptosis of primary cultured rat ovarian granulosa cells via its increase in extracellular HMGB1. Reprod Sci. 2015;22(3):271–7.

72. Du XH, Zhou XL, Cao R, Xiao P, Teng Y, Ning CB, et al. FSH-induced p38-MAPK-mediated dephosphorylation at serine 727 of the signal transducer and activator of transcription 1 decreases Cyp1b1 expression in mouse granulosa cells. Cell Signal. 2015;27(1):6–14.

73. Liu J, Tian Y, Ding Y, Heng D, Xu K, Liu W, et al. Role of CYP51 in the regulation of T3 and FSH-induced Steroidogenesis in female mice. Endocrinology. 2017;158(11):3974–87.

74. Patel SS, Beshay VE, Escobar JC, Suzuki T, Carr BR. Molecular mechanism for repression of 17alpha-hydroxylase expression and androstenedione production in granulosa cells. J Clin Endocrinol Metab. 2009;94(12):5163–8.

75. Nogueira D, Cortvrindt R, Everaerdt B, Smitz J. Effects of long-term in vitro exposure to phosphodiesterase type-3 inhibitors on follicle and oocyte development. Reproduction. 2005;130(2):177–86.

76. Thomas RE, Thompson JG, Armstrong DT, Gilchrist RB. Effect of specific phosphodiesterase isoenzyme inhibitors during in vitro maturation of bovine oocytes on meiotic and developmental capacity. Biol Reprod. 2004; 71(4):1142–9.

77. Assou S, Haouzi D, Mahmoud K, Aouacheria A, Guillemin Y, Pantesco V, et al. A non-invasive test for assessing embryo potential by gene expression profiles of human cumulus cells: a proof of concept study. Mol Hum Reprod. 2008;14(12):711–9.

78. Hamel M, Dufort I, Robert C, Gravel C, Leveille MC, Leader A, et al. Identification of differentially expressed markers in human follicular cells associated with competent oocytes. Hum Reprod. 2008;23(5):1118–27.

79. Racowsky C, Needleman DJ. Cumulus cell gene expression as a potential biomarker for oocyte quality. Fertil Steril. 2018;109(3):438–9.

80. Fuchs Weizman N, Wyse BA, Gat I, Balakier H, Sangaralingam M, Caballero J, et al. Triggering method in assisted reproduction alters the cumulus cell transcriptome. Reprod Biomed Online. 2019;39(2):211–24.

81. Dobin A, Davis CA, Schlesinger F, Drenkow J, Zaleski C, Jha S, et al. STAR: ultrafast universal RNA-seq aligner. Bioinformatics. 2013;29(1):15–21.

82. Love MI, Huber W, Anders S. Moderated estimation of fold change and dispersion for RNA-seq data with DESeq2. Genome Biol. 2014;15(12):550.

83. Merico D, Isserlin R, Stueker O, Emili A, Bader GD. Enrichment map: a network-based method for gene-set enrichment visualization and interpretation. PLoS One. 2010;5(11):e13984.

84. Ovarian Kaleidoscope Database. 1999. Available from: http://okdb.appliedbioinfo.net/. Accessed 17 Jul 2020.

85. Livak KJ, Schmittgen TD. Analysis of relative gene expression data using real-time quantitative PCR and the 2 − ΔΔCT method. Methods. 2001;25(4): 402–8.

The original article [1] has been corrected.
